# Distinct sets of PIWI proteins produce arbovirus and transposon-derived piRNAs in *Aedes aegypti* mosquito cells

**DOI:** 10.1093/nar/gkv590

**Published:** 2015-06-11

**Authors:** Pascal Miesen, Erika Girardi, Ronald P. van Rij

**Affiliations:** Department of Medical Microbiology, Radboud University Medical Center, Radboud Institute for Molecular Life Sciences, P.O. Box 9101, 6500 HB Nijmegen, The Netherlands

## Abstract

The PIWI-interacting RNA (piRNA) pathway is essential for transposon silencing in many model organisms. Its remarkable efficiency relies on a sophisticated amplification mechanism known as the ping-pong loop. In Alphavirus-infected *Aedes* mosquitoes, piRNAs with sequence features that suggest ping-pong-dependent biogenesis are produced from viral RNA. The PIWI family in *Aedes* mosquitoes is expanded when compared to other model organisms, raising the possibility that individual PIWI proteins have functionally diversified in these insects. Here, we show that Piwi5 and Ago3, but none of the other PIWI family members, are essential for piRNA biogenesis from Sindbis virus RNA in infected *Aedes aegypti* cells. In contrast, the production of piRNAs from transposons relies on a more versatile set of PIWI proteins, some of which do not contribute to viral piRNA biogenesis. These results indicate that functional specialization allows distinct mosquito PIWI proteins to process RNA from different endogenous and exogenous sources.

## INTRODUCTION

In the animal kingdom, three major classes of small silencing RNAs exist: microRNAs (miRNAs), small interfering RNAs (siRNAs) and PIWI-interacting RNAs (piRNAs) (1). All of these function in the context of proteins from the Argonaute superfamily. siRNAs and miRNAs associate with the AGO clade, whereas piRNAs are bound by the PIWI clade of Argonaute proteins (2). The small RNAs guide these proteins to complementary RNA molecules, which typically results in sequence-dependent suppression of those targets. Some Argonaute proteins can cleave their target RNAs (slicer activity), which are then susceptible to degradation by cellular exonucleases (3). PIWI proteins, however, are an exception, since their cleavage products can be processed into new piRNAs (4–7).

In animals, the piRNA pathway is key to the protection of the genome against the activity of transposable elements (TEs) (8,9). Still, our knowledge of piRNA biogenesis is incomplete and remains limited to a few model organisms. In the fruit fly *Drosophila melanogaster*, piRNA biogenesis involves two mechanisms: the primary processing pathway and a secondary amplification pathway, referred to as the ping-pong loop (10). The primary pathway generates from genomically encoded precursors a pool of primary piRNAs, which are loaded into the PIWI proteins Piwi and Aubergine (Aub) (4). From this initial piRNA collection, the ping-pong loop selectively amplifies Aub-bound piRNAs that recognize transcripts of active transposons (4,5). The PIWI protein Ago3 engages in this sophisticated feed-forward mechanism along with Aub. Both proteins mutually produce the piRNA precursors for each other, since the 3′ cleavage products generated by Aub can be transferred to Ago3 and vice versa (4,5,11,12). Once loaded in a PIWI protein, piRNA precursors are further processed into mature piRNAs, which are 25–30 nt in size and contain a 2′-O-methyl group at their 3′ terminal nucleotide (8). Aub-bound piRNAs commonly start with a uridine (1U) and, since target slicing by PIWI proteins occurs between nucleotide 10 and 11, the complementary Ago3-bound piRNAs typically have a 10 nt overlap and contain an adenine at position 10 (10A) (4,5). This specific sequence signature is a hallmark of piRNAs that have been amplified by the ping-pong loop. piRNA amplification was initially thought to occur exclusively in germline tissues, but recently, piRNAs have been detected in somatic cells in several organisms, including various mosquito species (13–16).

Blood-sucking mosquitoes are crucial for the transmission of many arthropod-borne viruses (arboviruses). Intriguingly, infected mosquitoes generally do not show signs of pathology, suggesting that they possess efficient pathways to resist or tolerate virus infection (17). Key to antiviral immunity in insects is the RNA interference (RNAi) pathway with at its core 21 nt viral siRNAs (vsiRNAs) bound to Argonaute 2 (Ago2) (18,19). These vsiRNAs are processed from viral double-stranded RNA (dsRNA), which accumulates in infected cells during the replication cycle of many viruses (20). Unexpectedly, besides vsiRNAs, we and others have recently cloned and sequenced viral small RNAs with the sequence signature of ping-pong-dependent piRNAs in somatic cells of infected *Aedes* mosquitoes and in cell lines derived from these insects (14,15,21–23). Still, the biogenesis and function of these viral piRNAs (vpiRNAs) are not well understood. Neither has their association with a PIWI protein been demonstrated, which would formally classify these viral small RNAs as *PIWI interacting* RNAs. Interestingly, whereas flies encode three PIWI proteins, the PIWI family is expanded to eight members (Piwi1–7 and Ago3) in *Aedes aegypti*. However, with the exception of Ago3, no 1:1 orthology exists between *Aedes* PIWI proteins and known piRNA biogenesis factors (24). Combined knockdown of all *Aedes* PIWI proteins abrogates vpiRNA biogenesis (21), but the contribution of the individual PIWI proteins to vpiRNA biogenesis in mosquitoes remains obscure.

The diversification of PIWI proteins and the accumulation of ping-pong-dependent vpiRNAs suggest that the PIWI pathway in mosquitoes has gained additional functions besides the repression of transposon activity. An exciting possibility is that the PIWI gene expansion has allowed functional specialization in producing piRNAs from different RNA sources. Here, we test this hypothesis making use of the piRNA competent *Aedes aegypti* Aag2 cell line. These cells produce Alphavirus-derived piRNAs with striking similarities to vpiRNAs in the adult mosquito (14). In addition, their PIWI protein repertoire strongly mimics the PIWI expression profile in somatic tissues of adult mosquitoes, as recently determined by RNA sequencing (25). Therefore, the Aag2 cell line is an accessible and relevant model system to investigate the molecular mechanisms of (viral) piRNA biogenesis in *Aedes*. Using this model, we identify Piwi5 and Ago3 as the core proteins of the mosquito ping-pong loop. During infection with Sindbis virus (SINV), the production of piRNAs of viral origin is almost exclusively dependent on ping-pong amplification by Piwi5 and Ago3, whereas the biogenesis of transposon-derived piRNAs is more versatile and involves additional members of the PIWI protein family. These data suggest that specialized arms of the mosquito PIWI pathway engage in piRNA biogenesis from endogenous or exogenous RNAs.

## MATERIALS AND METHODS

### Transfection and infection of Aag2 cells

For immunoprecipitation (IP) and immunofluorescence analyses (IFA), Aag2 cells were transfected with expression plasmids encoding individual PIWI proteins and, where indicated, infected with SINV at a Multiplicity of Infection (MOI) of 1 immediately after transfection. For knockdown experiments, Aag2 were transfected with dsRNA and re-transfected 48 h after the first transfection to boost the knockdown. Where indicated, cells were then infected with SINV at an MOI of 1. Unless stated differently, samples were harvested 48 h post infection. For a detailed description of the experimental procedure, the cloning of expression plasmids, cell culture conditions and virus production, see Supplementary data.

### Northern blotting and qPCR

Small RNA northern blotting was performed using 1-ethyl-3- (3-dimethylaminopropyl) carbodiimide (Sigma) crosslinking after size separation on polyacrylamide gels as detailed in (26). For high molecular weight northern blot, RNA was separated on agarose gels and crosslinked using UV irradiation. For quantitative RT-PCR (RT-qPCR), total RNA was RNase treated, reverse transcribed, and PCR amplified in the presence of SYBR green. For a detailed description of the experimental procedures, the sequences of the northern blot probes and the qPCR primers, see Supplementary data.

### Western blotting and immunofluorescence analysis

For western blotting, proteins were separated on polyacrylamide gels, blotted to nitrocellulose membranes and probed with the indicated antibodies. IFA were performed on paraformaldehyde-fixed and permeabilized Aag2 cells. For a detailed description of the experimental procedure and the antibodies, see Supplementary data.

### IP

Lysates from Aag2 cells expressing V5–3xFlag tagged PIWI proteins were pre-cleared with protein G agarose beads and then incubated with V5-agarose beads (Sigma). The immunoprecipitates were washed, and RNA was isolated from the beads for subsequent analyses. For a detailed description of the experimental procedure, see Supplementary data.

### Cytoplasmic and nuclear fractionation

Aag2 cells were lysed in cytoplasmic lysis buffer (25 mM Tris HCl, pH 7.5, 50 mM NaCl, 2 mM EDTA, 0.5% NP40, 1x protease inhibitors) and the cytoplasmic fraction was separated from the nuclear pellet by centrifugation. The nuclear pellet was washed in cytoplasmic lysis buffer and lysed in 1x SDS PAGE loading buffer for protein analysis or Isol-RNA lysis reagent (5 PRIME) for RNA isolation. Similarly, 5x SDS PAGE loading buffer or Isol-RNA lysis reagent was added to the cytoplasmic fraction for further processing. Protein or RNA fractions representing an equal number of cells were loaded on gel for western or northern blot analyses, respectively.

### Preparation of small RNA libraries and bioinformatic analyses

For the analysis of small RNAs in PIWI protein knockdown samples, small RNA libraries were prepared as previously described (27) and sequenced on an Illumina HiSeq 2500. The sequence data were analyzed with Galaxy (galaxyproject.org) (28). Reads were clipped from the adapter sequence and mapped with Bowtie, version 1.1.2 (29), to the SINV genome (pTE2J-3′ GFP) or to the *Aedes aegypti* transposon database (http://tefam.biochem.vt.edu; sequences downloaded on April 10, 2014). Size profiles of the small RNAs were obtained from all reads that mapped to these sequences with a maximum of one mismatch. Read counts were normalized to the size of the corresponding library and expressed as ‘% of library’. To analyze the genome distribution of vpiRNAs or vsiRNAs, the 5′ ends of the 25–30 nt or 21 nt SINV-mapping reads were plotted onto the viral genome. For plotting the genome distribution of vpiRNA reads from the PIWI IPs, the number of reads in the GFP-IP was subtracted from the PIWI-protein IP, to correct for background binding. When this corrected normalized read count was a negative value, it was set to zero. The overlap probability of viral piRNAs has been determined using the approach detailed in (30) using the small RNA signature tool available at the Mississippi Galaxy instance (mississippi.fr). Sequence logos were generated using WebLogo3.3 (31,32) using the tool available at the Galaxy main server. For analyzing the number of piRNAs that map to individual transposons, only uniquely-mapping reads were taken into consideration. For each transposons, the piRNA enrichment upon PIWI knockdowns relative to the luciferase control knockdown was calculated and hierarchical clustering of the transposons was performed using Multiple experiment viewer (version 4.8, Tm4) (33). Sequence data have been deposited in the NCBI Sequence Read Archive under accession number SRA188616.

## RESULTS

### Individual vpiRNAs are highly abundant in SINV-infected Aag2 cells

**Figure 1.**
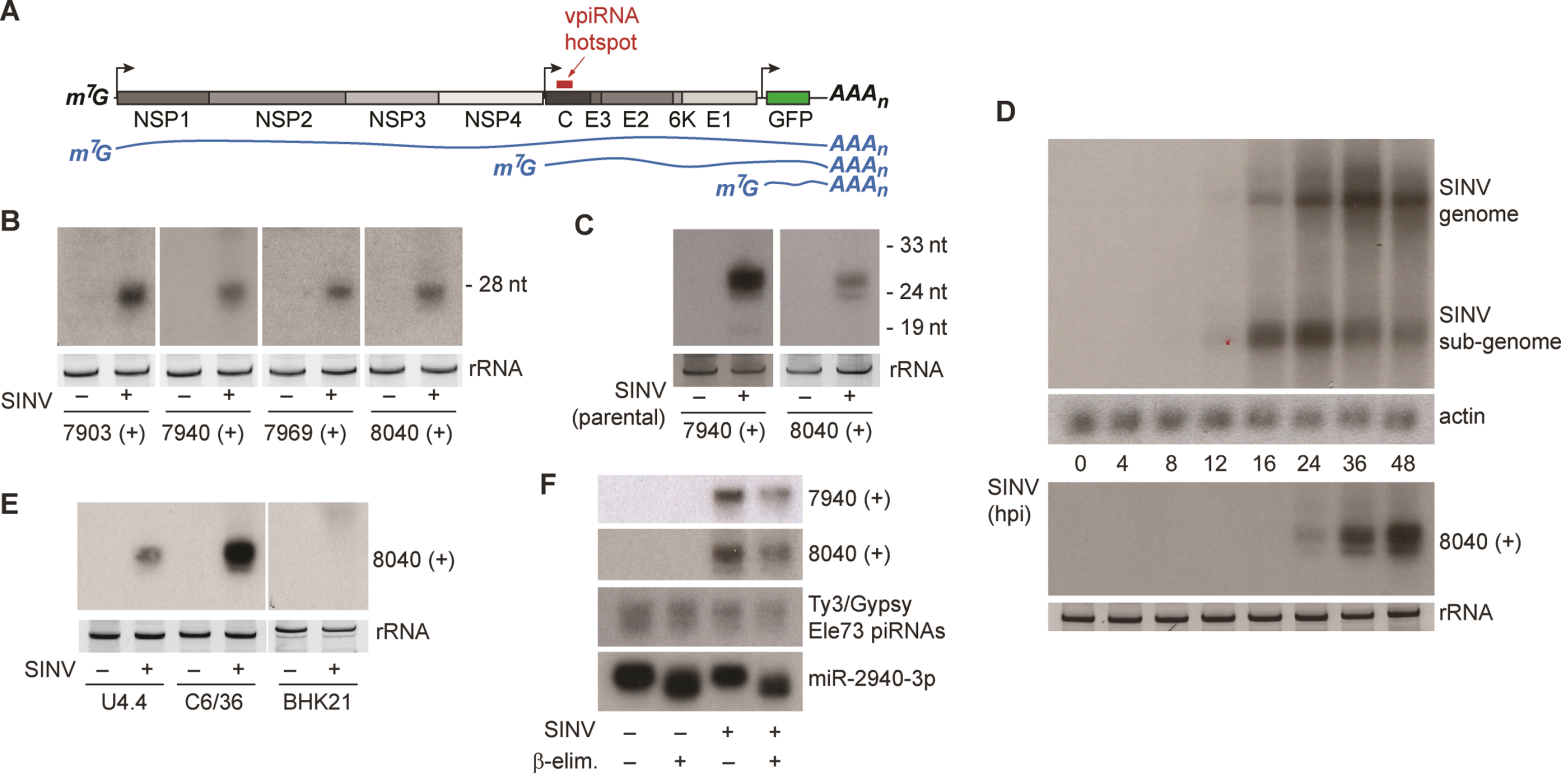
Selected mature vpiRNAs are abundant in Aag2 cells. (**A**) Schematic representation of the SINV-GFP genome. The individual viral proteins are indicated in gray and the position of the piRNA hotspot is marked by the red bar. The blue lines show the three (+) strand RNA species that can be found in infected cells. (**B**) Small RNA northern blot of four vpiRNAs in uninfected or SINV infected Aag2 cells. Probe names indicate the 5′ end position of the detected vpiRNAs, which are all derived from the SINV (+) strand. (**C**) Small RNA northern blot for vpiRNA in uninfected or SINV (parental virus) infected Aag2 cells. (**D**) Northern blot analysis of viral genomic and subgenomic RNA (upper panel) or vpiRNAs (lower panel) using a probe against vpiRNA 8040 (+). Probing for actin mRNA serves as loading control. (**E**) Northern blot analysis of vpiRNA in uninfected or SINV-infected *Aedes albopictus* mosquito cells (U4.4 and C6/36) and baby-hamster kidney cells (BHK21). For small RNA northern blots in panels B to E, ethidium bromide staining of ribosomal RNA (rRNA) serves as loading control. In panel B the loading controls for 7903 (+) and 7940 (+) are identical, since the same membrane was subsequently hybridized to these probes after harsh stripping in hot 0.1% SDS. (**F**) Northern blot detection of vpiRNAs, Ty3/Gypsy element 73 transposon piRNAs or miR2940–3p. Before blotting, β-elimination was performed on total RNA as indicated.

Previously, deep sequencing of small RNAs in infected Aag2 cells identified vpiRNAs derived from SINV, a positive (+) strand RNA virus of the genus Alphavirus within the *Togaviridae* family (15). During SINV replication, the viral (+) RNA strand serves as a template for the production of negative (−) strand RNA, which in turn is a template for the production of full-length genomic RNA as well as for a subgenomic RNA species. The vast majority of vpiRNAs is derived from the viral (+) strand and has a 10A nucleotide bias, suggesting that their production requires ping-pong amplification. An ∼200 nt large hotspot region for vpiRNA biogenesis is located in the capsid gene, 300 nt downstream of the SINV subgenomic promoter (Figure 1A). Read counts of several vpiRNAs within this hotspot are similar to those of average to highly expressed miRNAs, suggesting that they are efficiently produced and stably retained in Aag2 cells.

We selected four highly abundant vpiRNA sequences from the subgenomic hotspot region for small RNA northern blotting, all of which derive from the viral (+) strand. Indeed, these vpiRNAs were readily detected by northern blot in SINV-infected Aag2 cells (Figure 1B). These analyses were performed with recombinant SINV that expresses Green Fluorescent Protein (GFP) from a second subgenomic promoter, which permits simple assessment of infections (Figure 1A). However, the same vpiRNA sequences were found in Aag2 cells infected with the parental virus, indicating that vpiRNAs are not an artifact of transgene expression from the second subgenomic promoter (Figure 1C). During the course of infection, vpiRNAs were visible as soon as 24 h post infection (hpi), when infection was fully established (Figure 1D). In addition, northern blotting detected vpiRNAs in SINV-infected *Aedes albopictus* U4.4 and C6/36 mosquito cells, in line with previous observations using deep-sequencing technology (Figure 1E) (15). The higher accumulation of vpiRNAs in C6/36 cells is likely caused by elevated viral RNA replication, due to a defect in the antiviral RNAi response in these cells (34). As expected, mammalian BHK-21 cells, which allow SINV replication to similarly high levels but are devoid of an active piRNA pathway, did not produce SINV-derived piRNAs (Figure 1E). To analyze whether the detected viral small RNAs were mature vpiRNAs, we performed sodium periodate (NaIO_4_) oxidation followed by β-elimination. This reaction uncovers potential modifications of the ribose at the 3′ end of RNAs as it removes the terminal nucleoside of unmodified RNAs, leaving a 3′ monophosphate behind (35). Mature piRNAs are 2′-O-methylated at their 3′ end, and are therefore protected against this treatment (36,37). This distinguishes them from animal miRNAs, which have no 3′ end modification and are therefore shortened by β-elimination. Northern blot of individual vpiRNAs showed that their electrophoretic mobility is unaffected by β-elimination, indicating that their 3′ end is 2′-O-methylated. Likewise, piRNAs derived from a Ty3/Gypsy transposon were equally insensitive to the treatment. As expected, a miRNA was shortened by the reaction and its electrophoretic mobility clearly changed after treatment (Figure 1F). Taken together, these data indicate that individual, 2′-O-methylated vpiRNAs accumulate to high levels in infected Aag2 cells.

### Knockdown of Piwi5 and Ago3 abolishes secondary vpiRNA biogenesis

**Figure 2.**
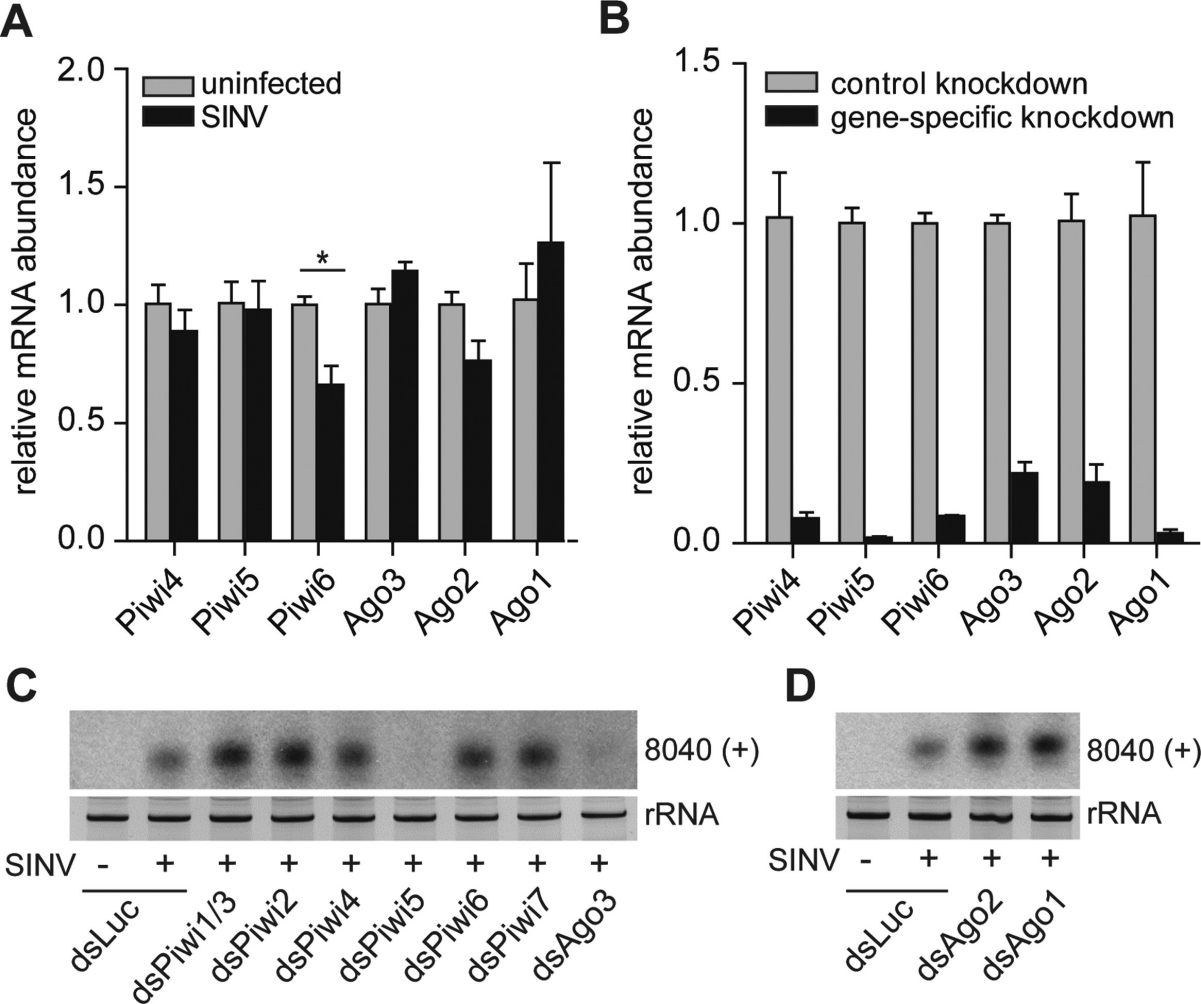
Piwi5 and Ago3 are required for secondary vpiRNA biogenesis. (**A**) qPCR analysis of the indicated PIWI/AGO transcripts in uninfected or SINV-infected Aag2 cells. Bars are the mean +/− SEM of three independent experiments. Student's t-test was used to determine statistical significance (*P < 0.05). (**B**) qPCR of PIWI/AGO transcripts in Aag2 cells at 48 h after transfection of control dsRNA (dsLuc) or dsRNA targeting the corresponding gene. Expression levels were normalized to the control knockdown. Bars are the mean +/− SEM of three independent experiments. All changes in mRNA abundance shown are statistically significant with *P* < 0.005. Expression of Piwi1–3 and Piwi7 were close to or below the detection limit and excluded from the analyses in panels A and B. (**C,D**) Northern blot for vpiRNA 8040 (+) upon knockdown of the indicated PIWI/AGO genes. Piwi1 and Piwi3 mRNA sequences are highly similar and are targeted by the same dsRNA. Staining of rRNA serves as loading control.

In Aag2 cells, transcripts of Piwi4, Piwi5, Piwi6 and Ago3 are readily detected; the abundance of Piwi1, Piwi2, Piwi3 and Piwi7, however, is considerably lower (15). This expression pattern mimics the PIWI expression profile in somatic tissue of adult mosquitoes, since Piwi1–3 are largely germline specific and Piwi7 is highly expressed only in the early embryo. (25). To investigate whether SINV infection alters PIWI mRNA abundance, we performed RT-qPCR for the individual PIWI transcripts, as well as for Ago1 and Ago2, which are involved in the biogenesis of miRNAs and siRNAs, respectively (38,39). Expression of Piwi1, Piwi2, Piwi3 and Piwi7 was close to or below the detection limit of our quantification method, both in uninfected and SINV-infected Aag2 cells. These genes were therefore excluded from qPCR analyses. With the exception of Piwi6, for which we noticed a mild reduction, infection with SINV did not substantially change mRNA expression of the remaining PIWI/AGO transcripts (Figure 2A). Next, we investigated which of the PIWI protein family members are involved in vpiRNA biogenesis. To this end, Aag2 cells were transfected with dsRNAs targeting the eight individual PIWI proteins (Piwi1–7/Ago3) prior to infection with SINV. Knockdown of Ago1 and Ago2 served as negative control. Using qPCR, we verified specific and efficient knockdown of at least 78% for all PIWI/AGO proteins (Figure 2B, Supplementary Figure S1A–D). We then analyzed the levels of vpiRNAs by small RNA northern blot. Knockdown of Piwi5 and Ago3 resulted in substantial loss of vpiRNAs, while knockdown of the other PIWI proteins did not lead to apparent reduction of vpiRNA levels (Figure 2C, Supplementary Figure S1E). As expected, knockdown of Ago1 or Ago2 likewise did not cause reduced vpiRNA accumulation (Figure 2D). These data identify Piwi5 and Ago3 as the first biogenesis factors for vpiRNA biogenesis in *Aedes aegypti*.

### Piwi5 and Ago3 are required for vpiRNA biogenesis

**Figure 3.**
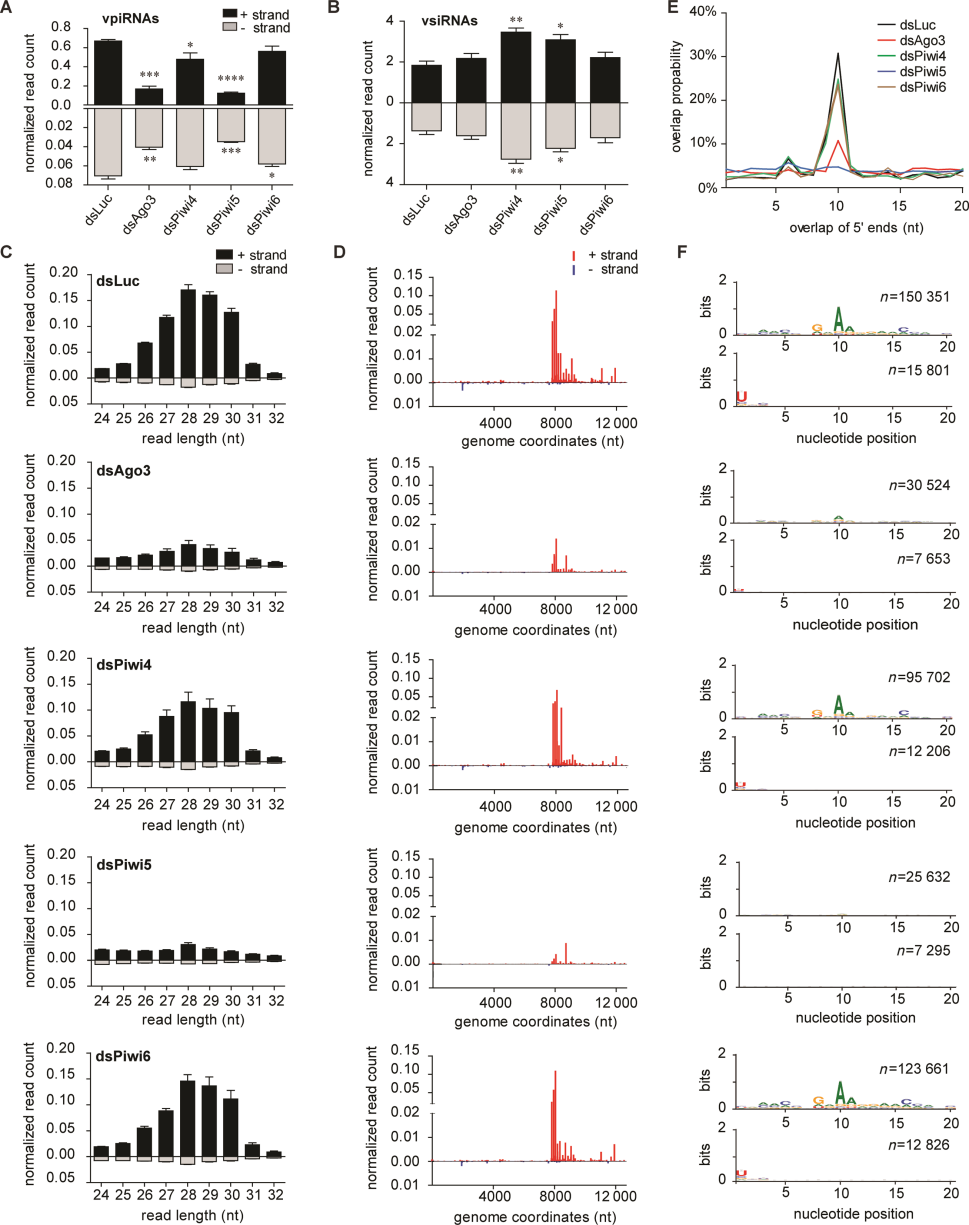
Piwi5 and Ago3 are required for vpiRNA biogenesis. (**A, B**) Number of 25–30 nt piRNA reads (A) and 21-nt siRNA reads (B) derived from the SINV (+) strand (black bars) and (−) strand (gray bars) in the indicated PIWI-protein knockdown libraries. Two-tailed student's t-test was used to determine statistical significance (*P < 0.05; **P < 0.01; ***P < 0.001: ****P < 0.0001). (**C**) Size profile of small RNAs mapping to the (+) strand (black bars) or the (−) strand (gray bars) of SINV. Bars in A–C are the mean +/− SEM of the three independent libraries. (**D**) Genome distribution of 25–30 nt small RNAs across the (+) strand (red) or (−) strand (blue) of the SINV genome. The average counts (three experiments) of the 5′ ends of the small RNA reads at each nucleotide position are shown. (**E**) The mean probability (*n* = 3) for 5′ overlaps between viral piRNAs from opposite strands in the indicated knockdown libraries. (**F**) Nucleotide bias at each position in the 25–30 nt small RNA reads mapping to the SINV (+) strand (upper panels) and (−) strand (lower panels). All reads of three independent experiments were combined to generate the sequence logo; *n*, number of reads.

Small RNA northern blotting is only suitable for the detection of highly abundant vpiRNAs which are, without exception, secondary piRNAs derived from the SINV (+) strand. To analyze the full repertoire of vpiRNAs, we prepared small RNA deep-sequencing libraries from SINV-infected Aag2 cells individually depleted of those PIWI proteins that are expressed in somatic tissues of adult mosquitoes and readily detectable in Aag2 cells (Piwi4, Piwi5, Piwi6 and Ago3). Knockdown of luciferase served as negative control. For each of these five conditions, three independent libraries were prepared and sequenced (Supplementary Table S1). Confirming our northern blot results, knockdown of Piwi5 and Ago3 resulted in considerable reduction of vpiRNAs, whereas knockdown of Piwi4 or Piwi6 only mildly affected vpiRNA levels (Figure 3A and C). In general, the vast majority of (+) strand vpiRNAs mapped to the subgenomic region of SINV, suggesting that the viral subgenome is the predominant source of secondary vpiRNAs. In contrast, the low number of (−) strand vpiRNAs mapped across the viral genome without enrichment at specific hotspot regions, suggesting that the entire (−) strand serves as a source for vpiRNAs. While the number of vpiRNAs was reduced upon Piwi5 and Ago3 knockdown, the genomic distribution of vpiRNAs did not change upon knockdown of any of the PIWI proteins (Figure 3D).

Loss of vpiRNAs could be explained by a reduced biogenesis rate or by suppressed virus replication, which would limit the amount of substrate RNA. However, the number of vsiRNAs produced in the different knockdown conditions remained stable or was even slightly elevated, arguing against the second option (Figure 3B, Supplementary Figure S2). To further confirm that the biogenesis of mature vpiRNAs is impaired in the absence of Piwi5 and Ago3 proteins, we analyzed the ping-pong signature of the remaining 25–30 nt small RNAs in the different knockdown conditions. Probing for 5′ end overlaps of sense and antisense small RNAs showed a strong reduction of read pairs with 10 nt overlaps upon knockdown of Ago3 and Piwi5 (Figure 3E). In addition, the characteristic 1U and 10A nucleotide bias of respectively antisense and sense piRNAs was lost upon Ago3 and Piwi5 knockdown (Figure 3F). In contrast, these hallmarks of ping-pong amplification were retained in the absence of Piwi4 and Piwi6 (Figure 3E and F). Collectively, these data underscore the pivotal role of Piwi5 and Ago3 in ping-pong-dependent biogenesis of SINV-derived piRNAs.

### Piwi5 and Ago3 bind piRNAs from opposite viral strands

We hypothesized that Piwi5 and Ago3 act as complementary partners of a ping-pong loop in *Aedes* mosquitoes. Such a model predicts that 1U-biased piRNAs derived from viral (−) strand would predominantly bind to one of the two PIWI proteins, whereas 10A-biased piRNAs from the (+) strand would associate with its counterpart (4,5). To test this hypothesis, we designed expression vectors for Piwi4, Piwi5, Piwi6 and Ago3 N-terminally fused to V5–3xFlag tags. As a control, we generated a V5–3xFlag-tagged GFP vector. Of note, multiple attempts to clone the Piwi5 cDNA failed, and using rapid amplification of cDNA ends (5′ RACE) we revised the current gene annotation (Supplementary Figure S3).

**Figure 4.**
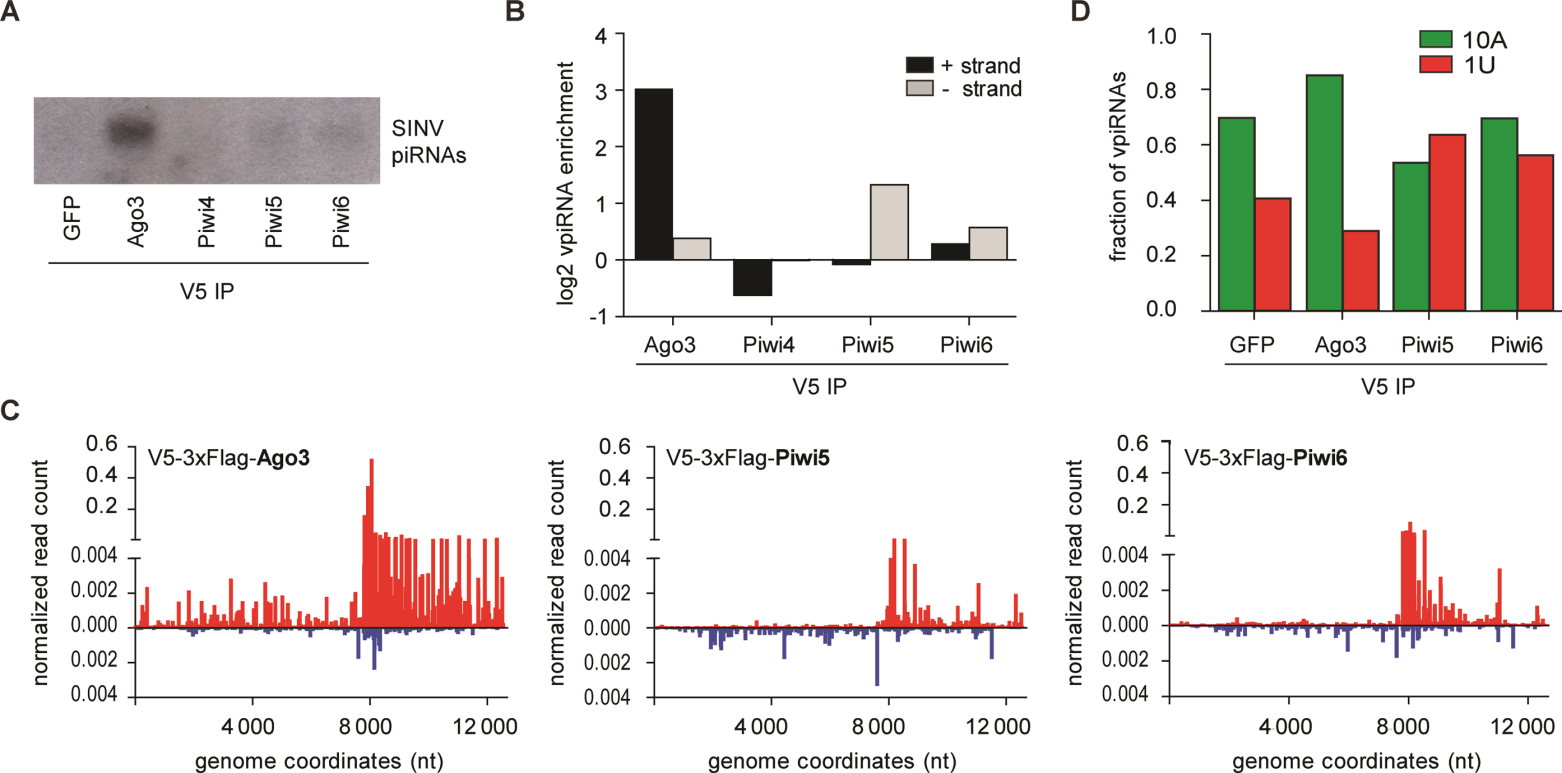
Association of vpiRNAs with individual PIWI proteins. (**A**) Northern blot analysis of vpiRNAs in RNA isolated from IPs of the indicated V5-epitope tagged proteins. Viral piRNAs were detected using a pool of the four probes presented in Figure 1A. (**B**) Enrichment of 25–30 nt small RNAs from the SINV (+) strand (black bars) or (−) strand (gray bars) in the IP of the indicated V5-epitope tagged PIWI proteins compared to the V5-tagged GFP-IP. (**C**) Distribution of 25–30 nt small RNAs in the indicated PIWI IPs across the (+) strand (red) or (−) strand (blue) of the SINV genome. Every data point shows the number of reads at each nucleotide position normalized against the size of the library (% of library). To account for background binding, the normalized read counts of the GFP-IP at each position were subtracted. (**D**) Fraction of 25–30 nt SINV-derived small RNA reads from the indicated deep-sequencing libraries that have an adenine at position 10 (10A; green bars) or uridine at position 1 (1U; red bars), respectively. No data for Piwi4 is shown in panels C and D since the V5-IP for this protein was not enriched for vpiRNAs.

We expressed the individual PIWI proteins in SINV-infected Aag2 cells and performed V5-ribonucleoprotein IP followed by vpiRNA northern blot. In line with our hypothesis, the 10A-biased vpiRNA sequences were enriched in Ago3 IP, but not in Piwi4–6 IPs (Figure 4A). These findings suggest that only Ago3 efficiently binds the highly abundant, (+) strand-derived vpiRNAs and that Piwi5, although required for their biogenesis, does not directly associate with this population of vpiRNAs. To analyze the PIWI association in more detail, we cloned and sequenced the small RNA fraction from Piwi4, Piwi5, Piwi6 and Ago3 IPs. As a control for non-specific binding, we sequenced small RNAs from a GFP-IP (Supplementary Table S1). Efficient IP was shown by the depletion of the transgenic proteins in the supernatant after IP (Supplementary Figure S4A). Confirming the northern blot analyses, (+) strand-derived vpiRNAs were strongly enriched in Ago3-IP only (Figure 4B, Supplementary Figure S4B). Similar to vpiRNAs sequenced from total RNA, Ago3-bound piRNAs were predominantly derived from the hotspot region downstream of the SINV subgenomic promoter (Figure 4C). In line with our hypothesis, Piwi5-IP exclusively enriched piRNAs derived from the SINV (−) strand (Figure 4B, Supplementary Figure S4D), which mapped across the entire length of the viral antigenome (Figure 4C). The Piwi4-IP was not enriched for vpiRNAs (Figure 4B, Supplementary Figure S4C) and Piwi6-IP was only mildly enriched for vpiRNAs predominantly from the viral (−) strand (Figure 4B, C and Supplementary Figure S4E).

Next, we analyzed the nucleotide bias of PIWI-protein associated vpiRNAs. To this end, we determined the fraction of 10A and 1U-containing vpiRNA reads in the PIWI-IPs that were enriched for vpiRNAs (Ago3, Piwi5 and Piwi6; Figure 4A and B). In the GFP control precipitation, 70% of the vpiRNA sequences had an adenine at position 10. This fraction increased to 85% in the Ago3-IP, but in none of the other PIWI-IPs (Figure 4D). Furthermore, the fraction of 1U-containing vpiRNAs declined from 40% in the GFP-IP to 29% in the Ago3-IP. Thus, parallel to raising the absolute number of (+) strand-derived vpiRNAs more than 8-fold (Figure 4B), Ago3-IP purified this population toward a stronger 10A nucleotide bias. In contrast, the Piwi5-IP was enriched for vpiRNAs with a uridine at position one (63%) and was depleted of 10A-containing sequences (53%), when compared to the control GFP-IP (Figure 4D). Piwi6-IP resulted in an enrichment of 1U-containing vpiRNAs (56%), which likely reflects the mild enrichment for (−) strand-derived vpiRNAs (Figure 4B). Altogether, these data formally classify the 25–30 nt SINV-derived small RNAs in Aag2 cells as *PIWI interacting* RNAs. In addition, our findings show that in *Aedes aegypti*, Ago3 and Piwi5 are the complementary core proteins of the ping-pong loop, which is the dominant mechanism for vpiRNA synthesis in response to SINV infection. Piwi4 and Piwi6, if at all, only have a minor contribution to vpiRNA biogenesis.

### Ago3 and Piwi5 co-localize with vpiRNAs in the cytoplasm

**Figure 5.**
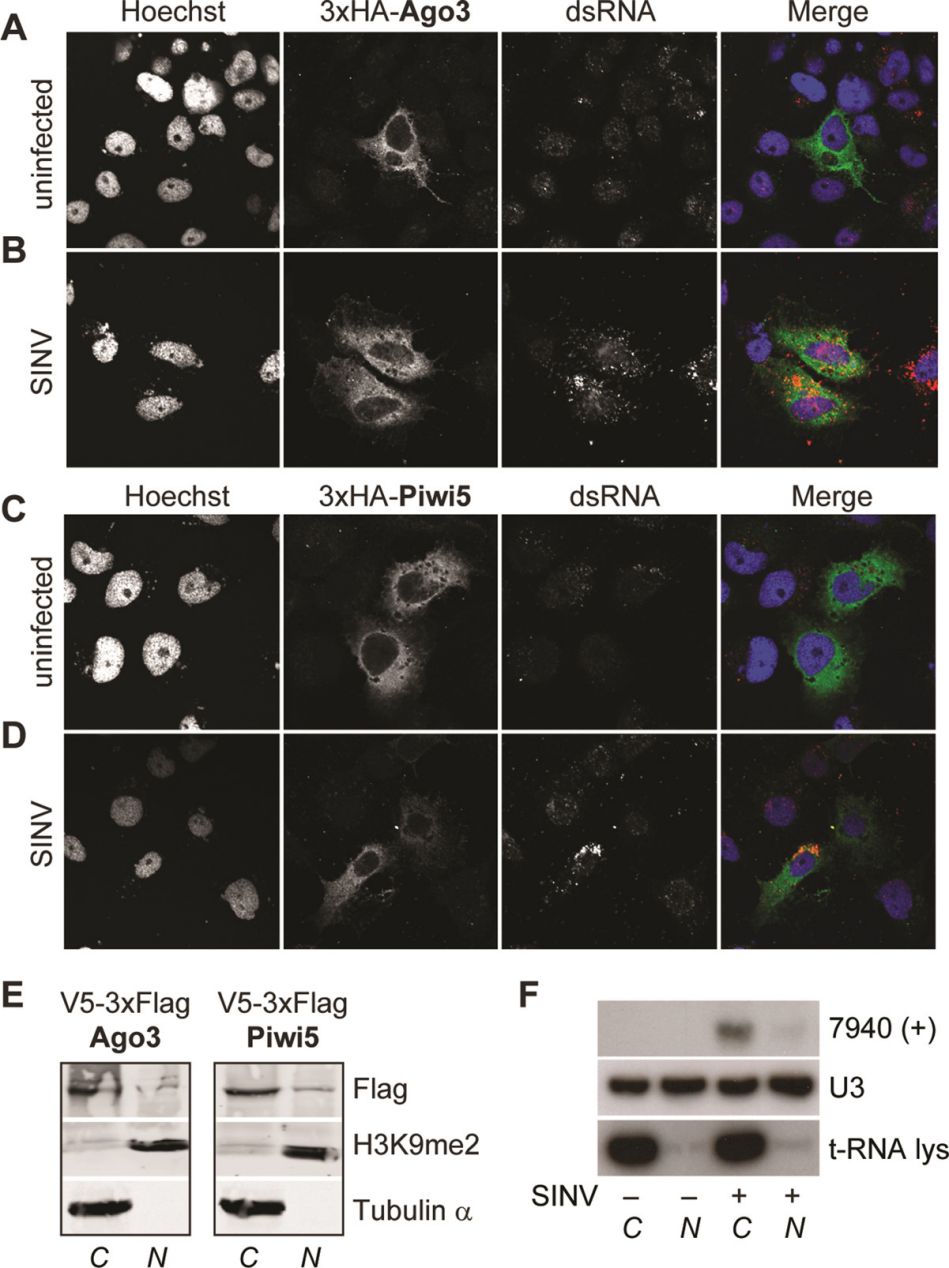
vpiRNA biogenesis occurs in the cytoplasm. Localization of 3xHA-tagged Ago3 (**A,B**) and Piwi5 (**C,D**) in uninfected (A,C) and SINV (parental virus) infected Aag2 cells (B,D) as determined by confocal microscopy. Hoechst staining indicates the nuclei. Infected cells were identified by a strong cytoplasmic dsRNA staining, which can be clearly distinguished from low-level background staining in non-infected cells. (**E**) Western blot analysis of V5–3xFlag-tagged Piwi5 and Ago3 in the cytoplasmic (*C*) and nuclear (*N*) fraction of Aag2 cell lysates. Tubulin and Histone 3 dimethylated at lysine 9 (H3K9me2) were used as cytoplasmic or nuclear markers, respectively. (**F**) Northern blot of vpiRNA 7940 (+) in nuclear and cytoplasmic fractions of Aag2 cells. U3 RNA and t-RNA lysine serve as nuclear and cytoplasmic marker, respectively.

In the *Drosophila* germline, ping-pong amplification of piRNAs occurs in a non-membranous perinuclear structure in the cytoplasm, termed *nuage*. In mutant flies with defects in Aub and Ago3 localization to this region, piRNA amplification is disrupted (40,41). Therefore, we analyzed the subcellular localization of 3xHA-tagged Piwi5 or Ago3 in Aag2 cells. Both proteins were diffusely expressed in the cytoplasm with only little expression in the nucleus (Figure 5A, C and Supplementary Figure S5A, C). In some instances, we found perinuclear enrichment for both proteins, but this was minor compared to the clear, ring-like localization of Aub and Ago3 in the *Drosophila* germline (4). SINV infection did not alter the subcellular localization of Piwi5 and Ago3. Furthermore, Piwi5 and Ago3 did not accumulate at sites of dsRNA production in infected cells (Figure 5B, D and Supplementary Figure S5B, D). The predominant expression of both Piwi5 and Ago3 in the cytoplasm was confirmed by western blotting after cytoplasmic and nuclear fractionation (Figure 5E). Thus, since SINV RNA replication occurs in the cytoplasm, viral RNAs and the vpiRNA core biogenesis factors are co-expressed in the cytoplasm. Indeed, the vast majority of vpiRNAs was also present in the cytoplasmic fraction (Figure 5F), suggesting that vpiRNA biogenesis occurs in the cytoplasm of infected Aag2 cells.

### Differential association of virus and TE-derived piRNAs with *Aedes* PIWI proteins

**Figure 6.**
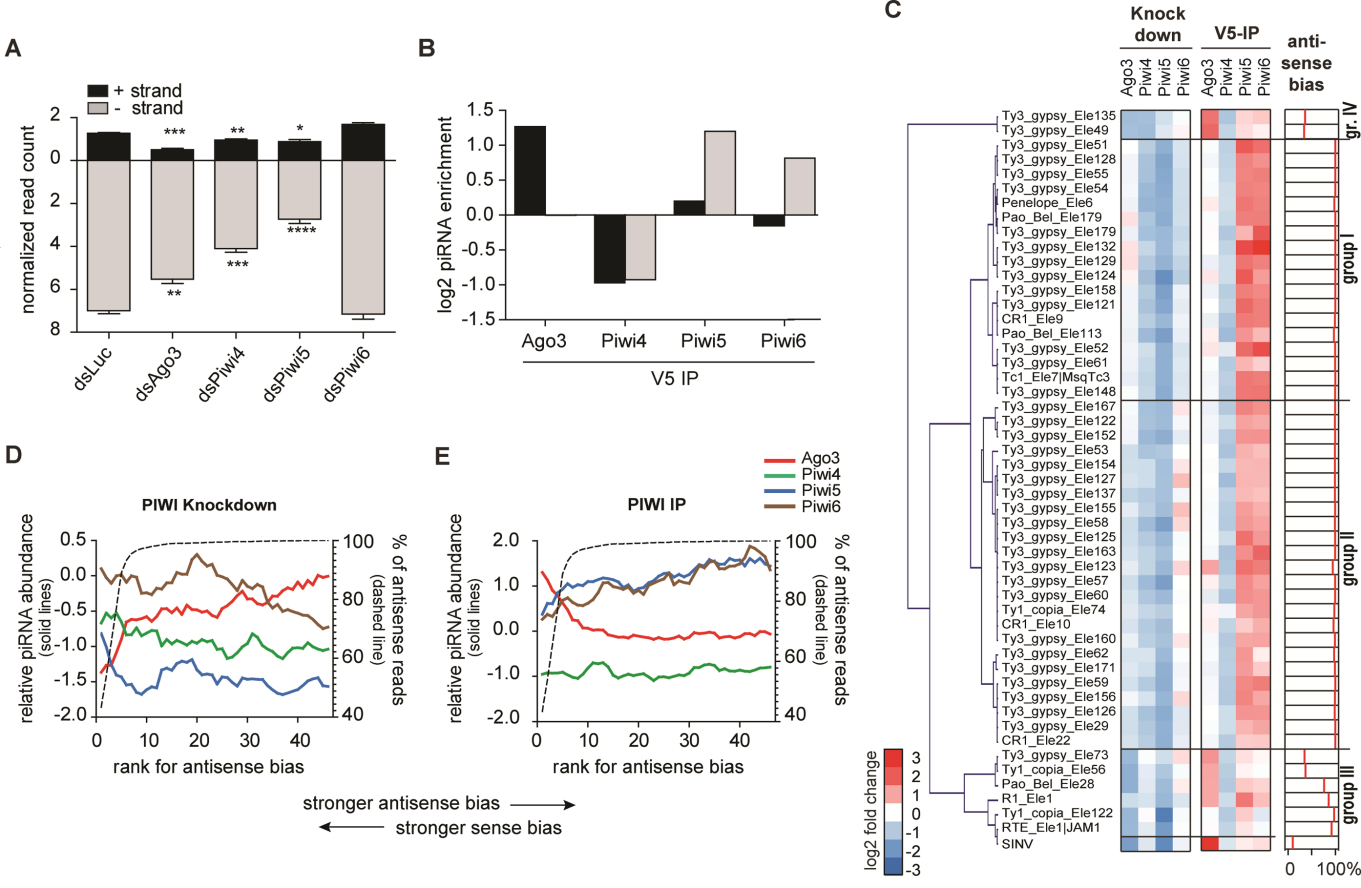
Association of TE-derived piRNAs with different PIWI proteins. (**A**) Normalized read counts of 25–30 nt reads from the different knockdown libraries mapping to the TEfam transposon database. The mean +/− SEM of three independent libraries are shown. Two-tailed student's t-test was used to determine statistical significance (*P < 0.05; **P < 0.01; ***P < 0.001; ****P < 0.0001). (**B**) Enrichment of 25–30 nt reads in the V5-IP of the indicated PIWI proteins compared to the GFP-IP. The number of reads from the (+) strand (black bars) or (−) strand (gray bars) in panels A and B was normalized to the corresponding library size. (**C**) Relative changes of the top 50 transposons upon PIWI protein knockdown and IP. Left panel: heat map showing the relative piRNA abundance in the indicated knockdown libraries compared to the control knockdown (dsLuc). These data were used to generate the hierarchical clustering. Middle panel: heat map showing the relative piRNA abundance in the indicated IP libraries over the control IP (GFP). Right panel: antisense bias, defined as the percentage of antisense 25–30 nt reads that uniquely map to the individual transposon sequences. (**D,E**) The 50 transposons from panel C were ranked according to their antisense bias. The mean relative piRNA abundance (log2-transformed) for five consecutive transposons is plotted with an offset of one rank number for the indicated knockdown libraries (D) or IP libraries (E). The corresponding antisense bias is indicated with the dashed line.

The expansion of the PIWI protein family in *Aedes aegypti* may have allowed functional specialization of PIWI proteins in the biogenesis of piRNAs from different sources, such as viral or transposon RNA. To test this hypothesis, we cataloged the requirement for individual PIWI proteins in the production of TE-derived piRNAs. We analyzed the repertoire of piRNAs that map to the annotated *Aedes aegypti* TE database (TEfam) upon PIWI protein knockdown. In line with previous observations (15), the vast majority of piRNAs was antisense to annotated TE sequences (Figure 6A, Supplementary Figure S6A). Furthermore, antisense TE-derived piRNAs had a strong 1U bias, whereas sense piRNAs showed a 10A bias, indicating the existence of a ping-pong-dependent piRNA population (Supplementary Figure S6B). However, whereas piRNA production from viral RNA was almost exclusively dependent on Ago3 and Piwi5, TE-derived piRNA levels were also decreased after Piwi4 depletion. Both upon knockdown of Piwi4 and, even more pronounced, upon knockdown of Piwi5 the number of antisense piRNAs was reduced. In contrast, Ago3 knockdown only mildly affected the levels of antisense TE-derived piRNAs, but caused the strongest reduction of sense strand piRNAs (Figure 6A). This suggests that, similar to the biogenesis of vpiRNAs, Ago3 might be directly involved in the production of (+) strand, 10A-biased TE-derived piRNAs. Indeed, when we analyzed the TE-derived piRNA populations in the different PIWI IPs, only the Ago3-IP was enriched for sense strand piRNAs. Strongest enrichment for antisense piRNAs, on the other hand, was observed in the Piwi5-IP (Figure 6B). Unexpectedly, although Piwi4 knockdown resulted in a decline of TE-derived piRNAs, the Piwi4-IP was depleted of, rather than enriched for transposon piRNAs (Figure 6B). This indicates that Piwi4 binds to neither viral nor TE-derived piRNAs, suggesting that the observed reduction of transposon piRNAs upon Piwi4 knockdown is likely to be an indirect effect that requires further investigation. Interestingly, although Piwi6 knockdown did not reduce transposon piRNA levels, Piwi6-IP was enriched for transposon piRNAs, albeit to a lower extent than the Piwi5-IP. It is currently unclear why knockdown of Piwi6 did not alter global transposon piRNA levels. Taken together, these data suggest that the requirement for different PIWI proteins is broader for TE-derived piRNAs than for SINV-derived piRNAs, production of which is solely dependent on Piwi5 and Ago3.

### piRNAs from individual TEs require different PIWI proteins for their biogenesis

Next, we analyzed the changes in piRNA levels for individual transposons upon knockdown of Piwi4–6 and Ago3. To classify transposons based on the PIWI proteins that medicate their piRNA biogenesis, we performed hierarchical clustering of the top 50 piRNA producing transposons. We identified four groups of transposons, based on the changes in piRNA abundance upon PIWI protein knockdown (Figure 6C). Group I and Group II transposons were characterized by a similar decrease of piRNAs upon knockdown of Piwi4 and Piwi5, but they differed in their dependence on Ago3 and Piwi6. Whereas piRNA biogenesis for group I transposons was reduced upon Piwi6 knockdown and not influenced by Ago3, group II transposons showed the opposite trend (Figure 6C, Supplementary Figure S6C and D). Group III transposons clustered with SINV, suggesting that piRNA biogenesis from these TE sequences depends on a similar set of PIWI proteins as vpiRNAs. Indeed, group III transposon piRNAs were reduced to a similar extent upon Piwi5 and Ago3 knockdown, but they were less affected by Piwi4 and Piwi6 knockdown (Figure 6C, Supplementary Figure S6E). This suggests that group III transposon piRNAs are, like vpiRNAs, produced in a ping-pong-dependent manner. Group IV is comprised of two transposons, which predominantly require Ago3 and Piwi4 for piRNA biogenesis (Figure 6C, Supplementary Figure S6F).

We next analyzed the association of the piRNAs from the selected 50 transposons with the four PIWI proteins. Reflecting our analyses of the total TE-derived piRNA population, Piwi4-IP was depleted of piRNAs from all individual transposons, indicating that it does not directly bind mature piRNAs. Piwi5 and Piwi6 were enriched for piRNAs from all groups of transposons. Yet, piRNA enrichment is strongest for group I and group II transposons and only weak for group III and group IV transposons (Figure 6C, Supplementary Figure S6G–J). Ago3-IP was enriched for piRNAs from group III and group IV transposons and an individual group II transposon (Ty3/Gypsy element 123). We noted that the piRNA population of group I and II TEs shows a strong antisense bias, whereas the piRNA population of group III and IV has a weaker antisense bias or even a slight sense bias. To further analyze this correlation, we sorted the transposons according to their antisense bias and performed a sliding window analysis on this ranking. Confirming our previous observations, Ago3 knockdown resulted in the strongest reduction of piRNA levels for transposons that have a sense or weak antisense bias and Ago3 dependence decreased with increasing antisense bias (Figure 6D). In line with these observations, Ago3-IP was only enriched for piRNAs from transposons that have strong sense bias (Figure 6E). Piwi5 knockdown generally had the biggest impact on piRNA levels, except for the transposons with the strongest sense bias (Figure 6D). Piwi6 knockdown primarily reduced piRNA levels of transposons with strong antisense bias, although the effect was minor compared to Piwi4 and Piwi5 knockdown (Figure 6D). Yet, Piwi6-IP was enriched for transposon piRNAs to a similar extent as Piwi5-IP, and both IPs tended to be more enriched for piRNAs from transposons with a strong antisense bias (Figure 6E). Since Piwi6-IP was almost not enriched for piRNAs of viral origin (Figure 4B), these data suggest that Piwi6 binds more specifically to piRNAs derived from selected transposons. Thus, whereas SINV piRNAs are almost exclusively produced via ping-pong amplification by Piwi5 and Ago3, TE-derived piRNA biogenesis directly or indirectly requires the activity of all analyzed PIWI proteins.

## DISCUSSION

Like in other invertebrates, recognition of viral dsRNA and its processing into vsiRNAs is key to antiviral immunity in mosquitoes (42). Yet, the recent discovery of vpiRNAs has challenged the idea that vsiRNAs are the sole small RNA species produced from viral RNA. Whereas the biogenesis of vsiRNAs is well-characterized in mosquitoes and fruit flies, little is known about the molecular mechanisms of vpiRNA production. The only cues come from the typical piRNA sequence signature that suggests a biogenesis pathway that includes ping-pong amplification (14,15,21–23).

Ping-pong amplification has previously been postulated for the production of TE-derived piRNAs in the fly (4,5). However, ping-pong-dependent piRNAs of viral origin have hitherto only been detected in mosquitoes and mosquito cells. In the fly, piRNA-sized viral small RNAs have been described in persistently infected ovarian somatic sheet (OSS) cells. These cells, however, are deficient of the secondary piRNA biogenesis factors Aub and Ago3 (11) and therefore vpiRNAs from OSS lack the ping-pong signature (43). In adult flies, PIWI proteins do not appear to be highly expressed in somatic tissues (4,5) and thus far no vpiRNA-like molecules have been identified in small RNA libraries of virus-infected flies. In sharp contrast, PIWI proteins are expressed in somatic cells of *Aedes aegypti* mosquitoes and secondary piRNAs can readily be detected outside the germline (14). Since most arboviruses exclusively infect somatic tissues and are not transmitted through the germline, it is likely that somatic PIWI expression has favored viral RNA as a new substrate for piRNA biogenesis.

*Aedes aegypti* Aag2 cells are competent in producing ping-pong-dependent vpiRNAs that have strikingly similar sequence features as vpiRNAs found in adult mosquitoes (14). Using this cell culture model we show that Ago3 and Piwi5 engage in a ping-pong amplification loop in which each of them binds vpiRNAs derived from opposite viral strands. Piwi5 predominantly binds 1U-biased, antisense piRNAs, whereas Ago3 preferentially associates with 10A-biased sense piRNAs, reflecting the nucleotide signature found for TE-derived piRNAs bound to *Drosophila* Aub and Ago3, respectively (4,5). These findings formally classify vpiRNAs as *PIWI interacting* RNAs. Somatic cells in adult *Aedes* mosquitoes express a strikingly similar set of PIWI proteins as Aag2 cells with only low expression of Piwi1–3 and Piwi7 (25). Piwi1 and Piwi3 are highly expressed specifically in the ovaries, a tissue that is generally not infected by SINV (44,45). Piwi7 is only expressed in the early embryo (25) and is therefore unlikely to contribute to the biogenesis of arbovirus-derived piRNAs. Thus, it is very likely that similar mechanisms are responsible for the production of SINV-derived piRNAs in Aag2 cells and adult mosquitoes.

The vast majority of vpiRNAs derives from the SINV (+) strand, has a 10A nucleotide bias and is associated with Ago3. Yet, the number of (+) strand, 10A-biased vpiRNAs is also strongly reduced upon Piwi5 knockdown. This is in line with the ping-pong model in which one PIWI protein generates the piRNA precursor for the other one. In the *Drosophila* germline, loss of function of Ago3 similarly eliminates the Aub-bound, antisense transposon-derived piRNA population (12). During SINV infection, vpiRNAs derived from the viral (−) strand accumulate to much lower levels, most likely because antigenomic RNA itself is scarce. Nevertheless, upon knockdown of Ago3 the number of antisense vpiRNAs declines even further, suggesting that in Aag2 cells the ping-pong loop is a full circle with both Ago3 and Piwi5 producing the piRNA precursors for each other.

It remains to be explained what determines the strand bias of Ago3-bound and Piwi5-bound vpiRNAs. In *Bombyx mori* Bmn4 cells, the MID–PIWI module of the PIWI proteins Siwi and Ago3 determines the strand bias of the associated piRNAs (46). The authors propose that the primary piRNA transcripts contain features that mark their nuclear origin and sort these precursors into Siwi based on the structure of the MID–PIWI domains. Since these transcripts tend to be antisense to transposon mRNAs, the nuclear origin of the piRNA precursor would dictate the strand bias of Siwi-associated piRNAs (46). Although this is an attractive model for transposon-derived piRNAs, it is unlikely to explain the strand bias of vpiRNAs, as it demands a nuclear component of the biogenesis pathway. We envision that vpiRNA production is a purely cytoplasmic event because SINV RNAs generally do not enter the nucleus. Thus, additional features must exist that sort piRNAs from the viral sense and antisense strands into Ago3 and Piwi5, respectively. The nature of such features is currently unknown. Likewise, it is not understood what discriminates the viral single-stranded RNA, which serves as piRNA precursor, from other abundant cellular mRNAs. Whereas dsRNA serves as an explicit non-self signal for the siRNA pathway, no such signal is known for the piRNA pathway.

*Aedes aegypti* is not a natural host for SINV, which is transmitted by *Culex* mosquitoes in the wild. To date, there is no conclusive data on whether *Culex* mosquitoes or cells derived from these animals produce Alphavirus-derived piRNAs. Yet, *Aedes* mosquitoes transmit Chikungunya virus (CHIKV), which belongs to the same virus family as SINV. Interestingly SINV and CHIKV produce ping-pong-dependent vpiRNAs with strikingly similar sequence features and genome distribution (14). The same is true for Semliki Forest virus (SFV), another member of the Alphavirus family (21) and probably CHIKV and SFV piRNA biogenesis relies on a similar, if not identical, molecular machinery as SINV. It is likely that specific features, common to Alphaviruses, are recognized by the piRNA biogenesis machinery and make the viral RNA a favorable piRNA substrate. These features must be independent of primary nucleotide sequence, since SINV, CHIKV and SFV only share little sequence similarity. Outside of the Alphaviruses, vpiRNAs with ping-pong signature have been shown for La Crosse virus (15), Rift Valley fever virus (22) and Schmallenberg virus (23), all of which belong to the *Bunyaviridae* family. In RNAi-deficient C6/36 cells, vpiRNAs from Dengue virus, a Flavivirus, have been proposed based on the small RNA size range and a 10A bias, but no 1U was detected (47). Additional studies did not detect Dengue virus-derived piRNA-sized small RNAs with the characteristic ping-pong signature (48,49). Future research will have to establish which viruses produce vpiRNAs and if the piRNA biogenesis mechanism is similar to the one described here.

The *Aedes aegypti* genome is remarkably rich in transposons (50), which are the dominant substrate for piRNAs in all studied model organisms. In *Aedes* mosquitoes, the diversification of the PIWI family may have facilitated the recognition of novel RNA substrates and even functional specialization of PIWI proteins in producing piRNAs from various RNA sources. Indeed, in Aag2 cells the biogenesis of SINV-derived piRNAs is abrogated specifically upon knockdown of Piwi5 or Ago3, but not Piwi4 or Piwi6. Knockdown of Piwi5 also causes a reduction in TE-derived piRNA levels for the vast majority of transposons, suggesting that it is essential for the biogenesis of both virus- and TE-derived piRNAs. Ago3, however, whereas crucial for vpiRNA biogenesis, is only relevant for piRNA production of transposons whose piRNAs are weakly antisense or sense biased. Thus, Ago3 may be dispensable for the biogenesis of primary piRNAs, an observation that needs validation in a full genetic Ago3 knockout. Interestingly, although nonessential for vpiRNA biogenesis, Piwi4 and Piwi6 do play a role in the production of piRNAs derived from a number of different TEs, suggesting functional specialization of PIWI proteins. Similar to Piwi5, Piwi6 associates with antisense piRNAs derived from a large number of transposon. Yet, Piwi6 knockdown does not greatly affect TE-piRNA levels. Thus far, the reason for this apparent contradiction is unknown. It may be explained by a dominant role of Piwi5 in binding (−) strand piRNAs, thereby veiling the effect of Piwi6 knockdown.

Amongst all the PIWI family members analyzed, Piwi4 did not directly bind piRNAs of either viral or transposon origin. In line with this observation, knockdown of Piwi4 results in a negligible decrease of SINV piRNA levels, which has previously been noted for a related virus (21). Interestingly, although devoid of piRNA binding capacity, knockdown of Piwi4 results in decreased TE-derived piRNA levels. This suggests that Piwi4 indirectly influences the production of transposon, but not SINV-derived piRNAs, by either modulating the activity of piRNA biogenesis factors or by influencing the amount of available substrate that could feed into the piRNA pathway. To our knowledge, the data presented here is the first example of functional specialization of PIWI proteins in producing piRNAs from endogenous or exogenous sources.

## Accession Number

NCBI Sequence Read Archive, SRA188616.

## SUPPLEMENTARY DATA

Supplementary Data are available at NAR Online.

SUPPLEMENTARY DATA
