# Supplementary material for: Distinct sets of PIWI proteins produce arbovirus and transposon-derived piRNAs in Aedes aegypti mosquito cells
Source: Nucleic Acids Res. 2015 Jun 11;43(13):6545–56. doi: 10.1093/nar/gkv590 (PMC4513867; doi:10.1093/nar/gkv590)
Supplement: SUPPLEMENTARY DATA [file supp_43_13_6545__index.html]

Distinct sets of PIWI proteins produce arbovirus and transposon-derived piRNAs in Aedes aegypti mosquito cells — Distinct sets of PIWI proteins produce arbovirus and transposon-derived piRNAs in Aedes aegypti mosquito cells — SUPPLEMENTARY DATA 

# Distinct sets of PIWI proteins produce arbovirus and transposon-derived piRNAs in *Aedes aegypti* mosquito cells

## SUPPLEMENTARY DATA

- SUPPLEMENTARY DATA
