# Supplementary material for: Distinct sets of PIWI proteins produce arbovirus and transposon-derived piRNAs in Aedes aegypti mosquito cells
Source: Nucleic Acids Res. 2015 Jun 11;43(13):6545–56. doi: 10.1093/nar/gkv590 (PMC4513867; doi:10.1093/nar/gkv590)
Supplement: SUPPLEMENTARY DATA [file supp_gkv590_nar-00950-a-2015-File008.pdf]

Supplementary Data

For

Distinct sets of PIWI proteins produce arbovirus and transposon-derived piRNAs in A*edes aegypti* mosquito cells.

Pascal Miesen[1], Erika Girardi[1], Ronald P. van Rij[1*]

[1]Department of Medical Microbiology, Radboud University Medical Centre, Radboud Institute for Molecular Life Sciences, P.O. Box 9101, 6500 HB Nijmegen, The Netherlands

*To whom correspondence should be addressed.

Tel: +31 24 3617574;

Mail: ronald.vanrij@radboudumc.nl

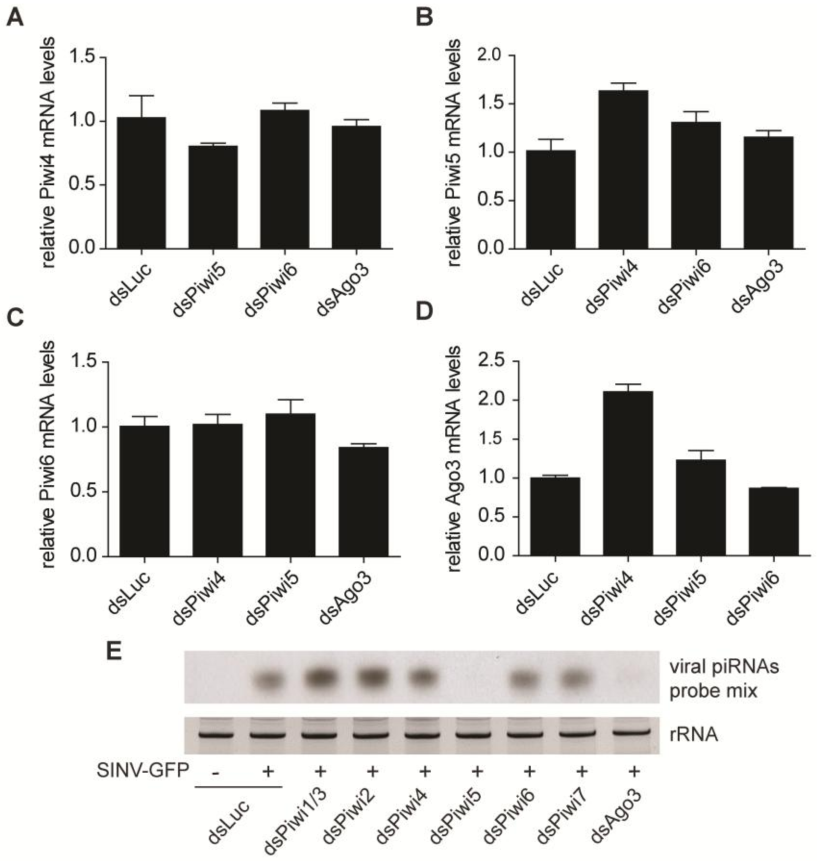

Figure S1: Specificity of PIWI knockdown (A-D) qPCR for Piwi4, Piwi5, Piwi6 and Ago3 transcripts, respectively, in Aag2 cells transfected with the indicated dsRNA. mRNA levels were normalized to the dsLuc control knockdown; bars represent the mean +/- SEM of three independent experiments. (E) Small RNA northern blot for SINV piRNAs in Aag2 cells transfected with the indicated dsRNA. A pool of four probes (individual probes shown in Figure 1A) was used to detect vpiRNAs. The rRNA loading control is identical to the one shown in Figure 2C, since the same membrane was subsequently hybridized to the individual 8040 (+) probe and the vpiRNA probe mix after harsh stripping.

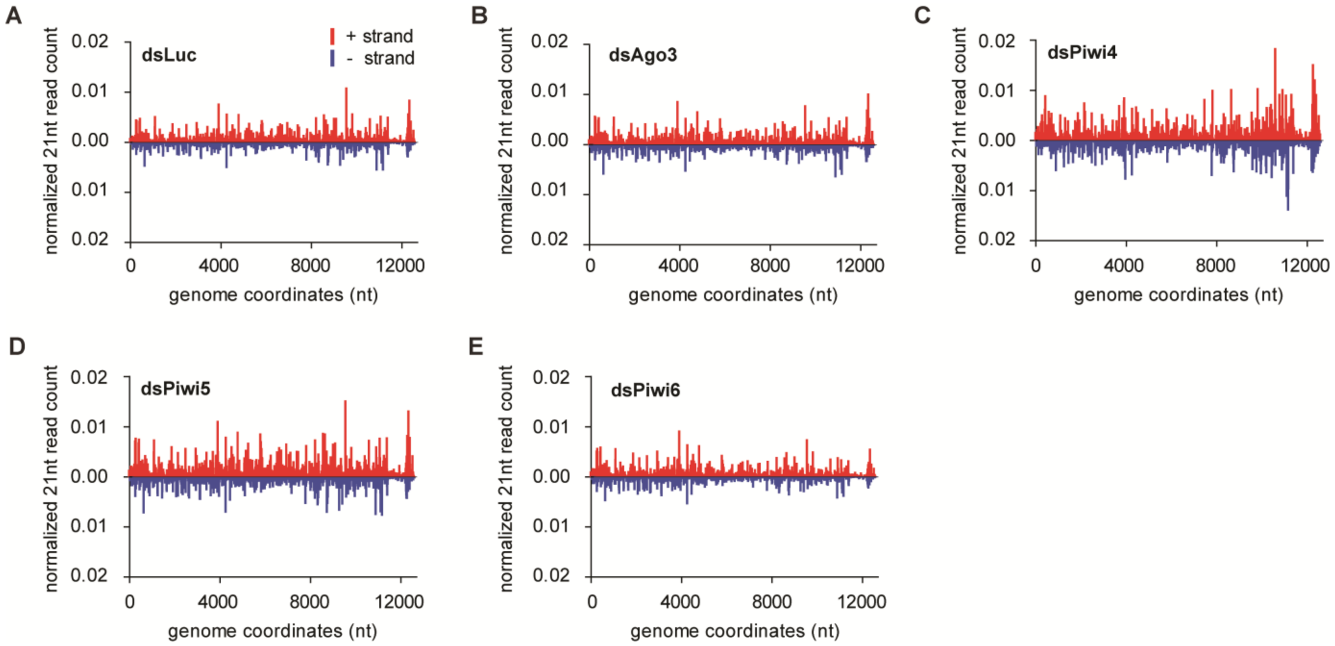

Figure S2: Levels of vsiRNAs remain stable upon PIWI knockdown (A-E) Genome distribution of 21 nt vsiRNAs across the (+) strand (red) or (-) strand (blue) of the SINV genome. The average counts (n=3) of the 5' ends of the small RNA reads at each nucleotide position are shown. The read number was normalized to the corresponding library size. The low read counts around position 12,000 coincides with the position of the GFP transgene, which is occasionally lost from the recombinant virus genome during virus replication.

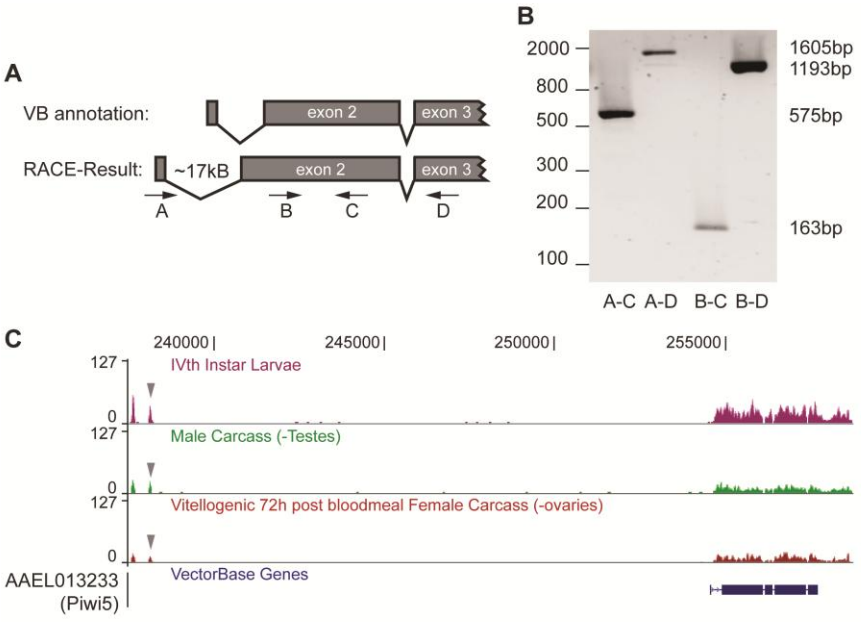

Figure S3: Revised gene annotation of Piwi5. (A) Schematic representation of the currently annotated gene topology of Piwi5 exon 1 to exon 3 published in Vectorbase (VB; release April 2015) and the revised gene structure as determined by 5' RACE on RNA isolated from Aag2 cells (exons and introns are not drawn to scale). The entire first annotated exon is not included in the Piwi5 mRNA. Instead, a previously un-annotated 108nt exon was found approximately 17 kB upstream of exon 2. Furthermore, the second exon includes 223 additional nucleotides at its 5' end. (B) Reverse-transcriptase PCR on RNA prepared from Aag2 cells. The positions of the primers are indicated by the arrows in panel A. A size marker is indicated on the left-hand side of the gel picture. Numbers on the right indicate the expected size of the PCR products. (C) Genome browser shots from *Aedes aegypti* supercontig 1.809 (www.vector.caltech.edu/). Numbers on top indicate the nucleotide position of the genomic scaffold. RNA sequencing data from the indicated tissue/developmental stage, as well as the annotated Piwi5 gene topology from Vectorbase are shown. The gray arrowheads indicate a putative, additional exon that we did not detect in our RACE analyses and subsequent cloning experiments.

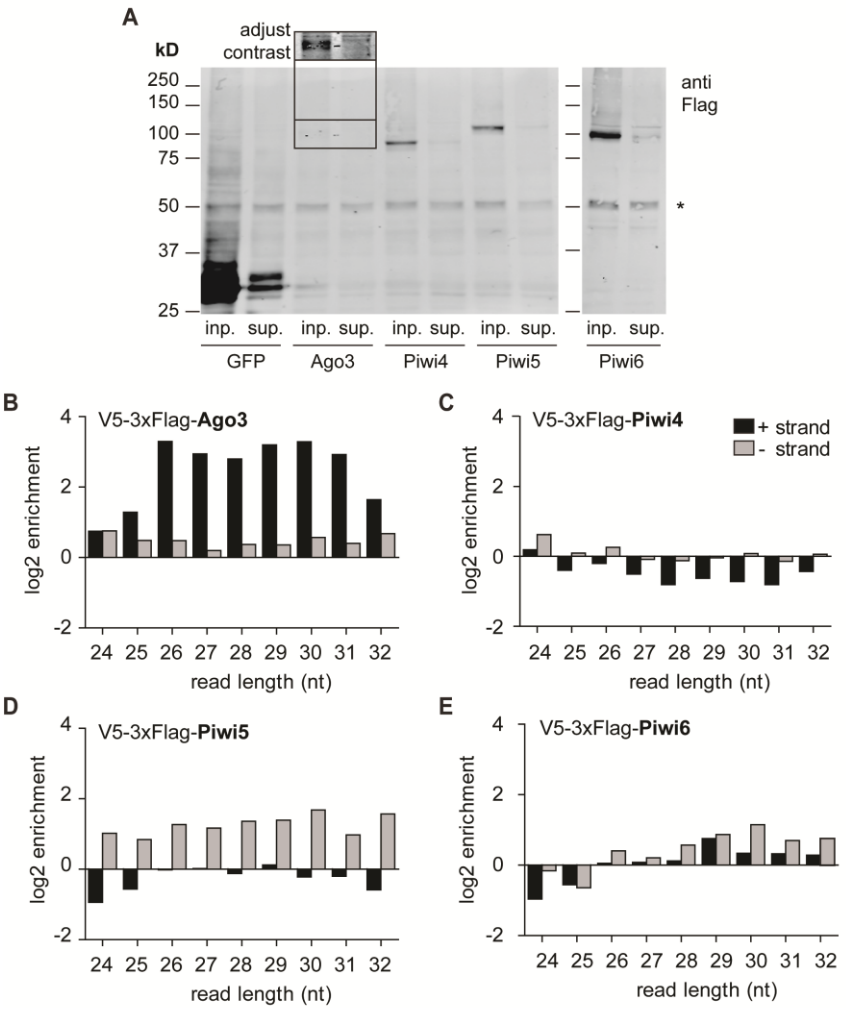

Figure S4: Fold enrichment of vpiRNAs in PIWI IPs. (A) Anti-Flag western blot for V5-3xFlag tagged PIWI proteins (size range 104kD-112kD) or V5-3xFlag-GFP (32.5kD). Protein expression is analyzed in 5% of the input (inp.) samples before V5-IP and 5% of the supernatant (sup.) after IP. * indicates a non-specific protein band. Linear contrast adjustment was used in Adobe Photoshop to enhance the signal for V5-3xFlag-Ago3, which was only lowly expressed. (B-E) Enrichment of small RNA reads in the V5-IPs for the indicated PIWI proteins over the control IP (V5-3xFlag-GFP IP). Log2-transformed enrichment scores of reads that map the SINV (+) strand and (-) strand are indicated in black and gray, respectively.

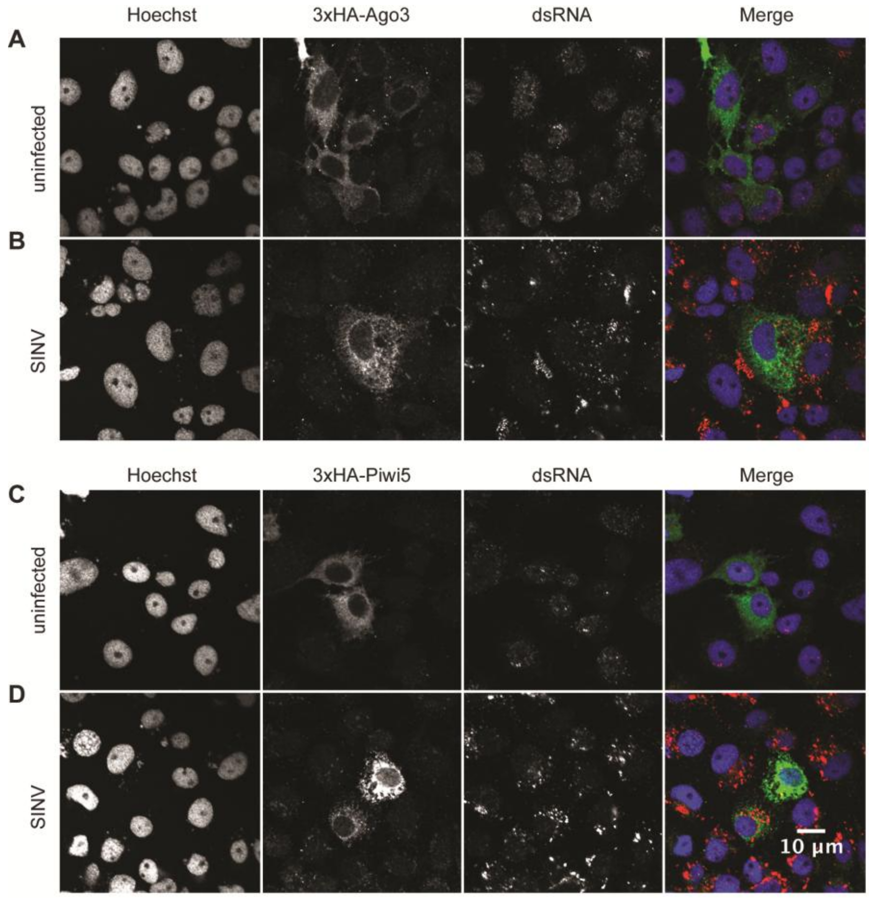

Figure S5: Ago3 and Piwi5 sub-cellular localization. Additional images of the experiment shown in Figure 5. The localization of 3xHA-tagged Ago3 (A,B) or Piwi5 (C,D) in uninfected (A,C) or SINV (parental virus) infected (B,D) Aag2 cells was determined by confocal immunofluorescence analysis. Hoechst staining was used to stain the nuclei. Intracellular dsRNA staining was used to identify infected cells as described in the legend to Figure 5. Scale bar indicates 10µm.

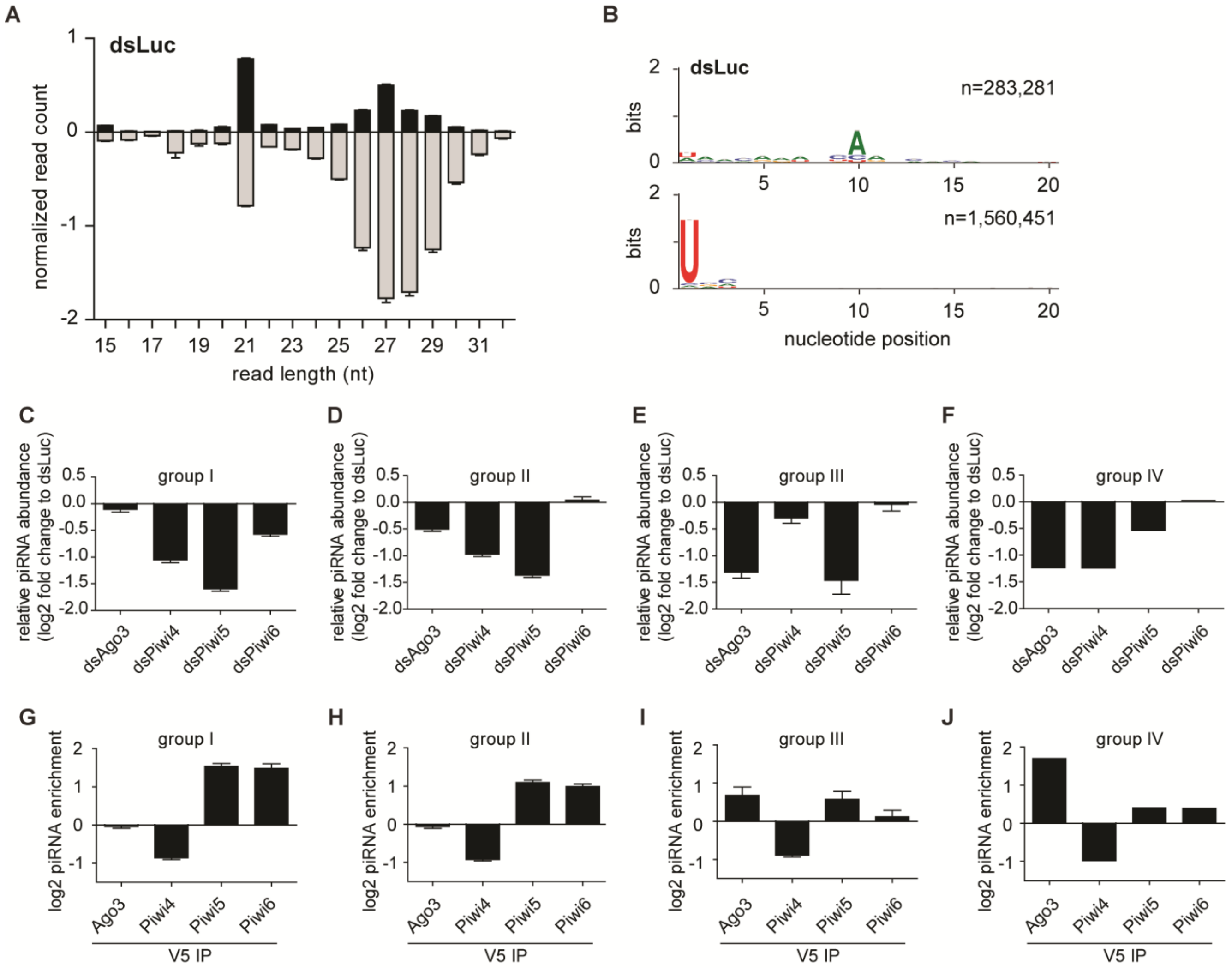

Figure S6: Groups of transposons can be classified based on their dependence on different PIWI proteins for piRNA biogenesis. (A) Size profile of small RNA reads from the dsLuc libraries that map to the collection of *Aedes aegypti* TE sequences published in the TEfam transposon database. Read counts were normalized to the corresponding library sizes. The average and SEM of the three independent libraries are shown. (B) Nucleotide bias at each position in the 25-30nt small RNA reads from the dsLuc libraries that map to the SINV (+) strand (upper panel) and (-) strand (lower panel). All reads of the three independent libraries were combined to generate the sequence logo; n, number of reads. (C-F) Relative abundance of 25-30nt reads in the indicated knockdown libraries compared to the dsLuc control libraries for group I (C), group II (D), group III (E) and group IV (F) transposons. (G-J) Enrichment of 25-30nt reads in the indicated IPs over the GFP control IP (for group I (G), group II (H), group III (I) and group IV (J) transposons. Panels C-J represent the mean of all transposons belonging to the corresponding group; error bars indicate the SEM. For a definition of group I to IV transposons, please consult Figure 6.

Table S1: Basic characteristics of the small RNA deep sequencing libraries.

For each category the number of reads is indicated. The numbers in brackets indicate the percentage of the total library size.

Library name	Total Library Size	SINV-GFP mappers	SINV-GFP mappers (25-30nt)	TEfam mappers	TEfam mappers (25-30nt)
Knockdown libraries					
**dsLuc-1**	8,037,253	411,612 (5.1%)	62,296 (0.8%)	913,974 (11.4%)	638,343 (7.9%)
**dsLuc-2**	8,250,209	442,710 (5.4%)	60,718 (0.7%)	995,007 (12.1%)	687,766 (8.3%)
**dsLuc-3**	6,071,409	229,474 (3.8%)	43,138 (0.7%)	729,563 (12.0%)	517,623 (8.5%)
**dsAgo3-1**	7,068,908	394,776 (5.6%)	12,497 (0.2%)	669,293 (9.5%)	403,034 (5.7%)
**dsAgo3-2**	7,904,410	437,717 (5.5%)	13,786 (0.2%)	767,388 (9.7%)	463,596 (5.8%)
**dsAgo3-3**	4,330,827	173,706 (4.0%)	11,894 (0.3%)	450,046 (10.4%)	283,516 (6.5%)
**dsPiwi4-1**	7,015,754	581,753 (8.3%)	44,936 (0.6%)	755,696 (10.8%)	366,409 (5.2%)
**dsPiwi4-2**	5,380,559	522,716 (9.7%)	30,809 (0.6%)	588,755 (10.9%)	248,328 (4.6%)
**dsPiwi4-3**	7,935,701	607,975 (7.7%)	32,163 (0.4%)	898,310 (11.3%)	423,123 (5.3%)
**dsPiwi5-1**	6,544,842	498,693 (7.6%)	9,524 (0.1%)	498,407 (7.6%)	206,713 (3.2%)
**dsPiwi5-2**	8,641,706	649,885 (7.5%)	12,565 (0.1%)	687,957 (8.0%)	301,385 (3.5%)
**dsPiwi5-3**	5,886,880	341,206 (5.8%)	10,838 (0.2%)	496,328 (8.4%)	245,686 (4.2%)
**dsPiwi6-1**	6,825,076	425,580 (6.2%)	43,645 (0.6%)	858,151 (12.6%)	586,311 (8.6%)
**dsPiwi6-2**	8,139,535	521,903 (6.4%)	41,781 (0.5%)	1,036,124 (12.7%)	688,963 (8.5%)
**dsPiwi6-3**	7,222,447	317,259 (4.3%)	51,061 (0.7%)	988,822 (13.7%)	682,842 (9.5%)
IP libraries					
**GFP**	1,374,646	19,542 (1.4%)	5,641 (0.4%)	137,252 (10.0%)	63,755 (4.6%)
**Ago3**	2,525,542	103,906 (4.1%)	74,696 (3.0%)	219,556 (8.7%)	138,592 (5.5%)
**Piwi4**	3,443,700	49,602 (1.4%)	9,828 (0.3%)	182,848 (5.3%)	83,766 (2.4%)
**Piwi5**	3,113,402	30,158 (1.0%)	14,803 (0.5%)	401,394 (12.9%)	310,650 (10.0%)
**Piwi6**	2,857,742	34,396 (1.2%)	14,760 (0.5%)	344,484 (12.1%)	263,350 (9.2%)

SUPPLEMENTARY MATERIALS AND METHODS

Cells and viruses

Aag2 cells were cultured at 25˚C in Leibovitz's L-15 medium (Invitrogen) supplemented with 10% heat inactivated Fetal calf serum (PAA), 2% Tryptose Phosphate Broth Solution (Sigma), 1x MEM Non-Essential Amino Acids (Invitrogen) and 50 U/ml penicillin and 50 μg/ml streptomycin (Invitrogen). U4.4 and C6/36 cells were cultured in the same medium at 28˚C. BHK-21 cells were cultured at 37˚C, 5% CO_2 in Dulbecco's modified Eagles Medium (DMEM) supplemented with 10% FCS and 50 U/ml penicillin and 50 μg/ml streptomycin. The virus used throughout this study, with the exception of Figure 1C, 5A-5D and S5, is a recombinant, double-subgenomic Sindbis virus expressing GFP from the second subgenomic promoter (pTE-3'2J-GFP). For Figure 1C, 5A-5D and S5, the parental virus was used (pTE-3'2J). Viruses were produced in BHK-21 cells as previously described (1). Unless stated differently, Aag2 cells were infected with SINV at a multiplicity of infection (MOI) of 1 for 48h hours.

Generation of plasmids and dsRNA production

Insect expression vectors, based on pAc5.1 (Invitrogen), were constructed for N-terminal tagging of proteins with V5-3xFlag or 3xHA tags. The full-length coding sequence of Piwi4, Piwi5, Piwi6 and Ago3 was amplified from Aag2 complementary DNA (cDNA) and cloned downstream of the tag sequences.

For dsRNA production, *in vitro* transcription using T7 RNA polymerase was performed on T7-promoter-flanked PCR products. To allow the formation of double-stranded RNA, the reaction products were heated to 80˚C and then gradually cooled to room temperature. Subsequently, the RNA was purified using the GenElute Mammalian Total RNA Miniprep Kit (Sigma) following the manufacturer's instructions. Primers used for plasmid or dsRNA production were:

XbaI-Piwi4	cagtctagaATGTCTGACCGTTACTCTC
NotI-Piwi4	catgcggccgcTTACAAGAAGTACAGCTTC
XbaI-Piwi5	cagtctagaATGGCGGATAGACAGCAAG
NotI-Piwi5	catgcggccgcTTACAGATAATAGAGTTTC
XbaI-Piwi6	cagtctagaATGGCTGATAATCCACAGG
NotI-Piwi6	catgcggccgcCTACAAAAAGTAAAGTTTC
XbaI-Ago3	cagtctagaATGTCCTCGCGGTTGAATTTAG
NotI-Ago3	catgcggccgcTCACAGGTAGAACAGTTT
T7Fw-Piwi1/3	taatacgactcactatagggagaCCACGCCCATCGTTTCAA
T7Re-Piwi1/3	taatacgactcactatagggagaCCTCAGTTTGTTCACCATA
T7Fw-Piwi2	taatacgactcactatagggagaCCGTCCTACTTTCCAGCAC
T7Re-Piwi2	taatacgactcactatagggagaGCGGCACTCCAGGGACAAT
T7Fw-Piwi4	taatacgactcactatagggagaCGTGGAAGTCCTTCTTCTCG
T7Re-Piwi4	taatacgactcactatagggagaTGTCAGTTGATCGCTTCTCAA
T7Fw-Piwi5	taatacgactcactatagggagaGCCATACATCGGGTCAAAAT
T7Re-Piwi5	taatacgactcactatagggagaCTCTCCACCGAAGGATTGAA
T7Fw-Piwi6	taatacgactcactatagggagaCAACGGAGGATCTTCACGAG
T7Re-Piwi6	taatacgactcactatagggagaAATCGATGGCTTGATTTGGA
T7Fw-Piwi7	taatacgactcactatagggagaGTGGAGGTCGTGGAGGTAAC
T7Re-Piwi7	taatacgactcactatagggagaGTTTGCGGTGTTTCCGTACT
T7Fw-Ago3	taatacgactcactatagggagaTGCTTACTCGTGTCGCGTAG
T7Re-Ago3	taatacgactcactatagggagaGGCATGGCAGATCCAATACT
T7Fw-Ago2	taatacgactcactatagggagaCTACGAGCAGGAGGTCAAGG
T7Re-Ago2	taatacgactcactatagggagaTCCATGCCTTTGAGGAAATC
T7Fw-Ago1	taatacgactcactatagggagaCCGGTCATCGAGTTCATGT

T7Re-Ago1	taatacgactcactatagggagaCGTGGCTTTGATCATGGTT
T7Fw-Luc	taatacgactcactatagggagaTATGAAGAGATACGCCCTGGTT
T7Re-Luc	taatacgactcactatagggagaTAAAACCGGGAGGTAGATGAGA

Transfection and infection of Aag2 cells

For IP or transgenic PIWI protein analysis, Aag2 cells were transfected with PIWI protein expression plasmids using X-tremeGENE HP (Roche) according to the manufacturer's instructions. Three hours post transfection, the medium was refreshed with supplemented Leibovitz's medium and, where indicated, infected with SINV. For knockdown experiments, Aag2 were transfected with dsRNA using X-tremeGENE HP. To increase knockdown efficiency, Aag2 cells were re-transfected at 48h after the first transfection. Three hours post transfection the medium was refreshed with supplemented medium and, where indicated, cells were infected with SINV. Unless stated differently, samples were harvested at 48 hours post infection.

Northern blot and β-elimination

Small RNA northern blot was performed as described previously (2). Briefly, total RNA was isolated using Isol-RNA Lysis Reagent (5 PRIME), size separated on a 15% PAGE gel, blotted to a nylon membrane (Hybond NX; Amersham) and cross-linked using 1-ethyl-3-(3-dimethylaminopropyl) carbodiimide (EDC; Sigma). For $NaIO_4$ oxidation and β-elimination, total RNA (in 13.5µl water) was added to 4µl borate buffer (148mM borax, 148mM boric acid, pH 8.6) and 2.5µl 200mM $NaIO_4$. After 10min incubation at room temperature, unreacted $NaIO_4$ was quenched by adding 2µl glycerol. After an additional 10min incubation at room temperature, samples were dried by centrifugation under vacuum and resuspended in 50µl of borax buffer (30mM borax, 30mM boric acid, 50mM NaOH, pH 9.5). Samples were incubated at 45˚C for 1.5h. Finally, RNA was ethanol-precipitated and reconstituted in 15µl water for further northern blot analysis. Hybridization with [^{32}P] labeled DNA oligonucleotides was performed overnight at 42˚C. The membrane was washed in 0.1% SDS, 2x SSC, followed by two washing steps in 0.1% SDS, 1x SSC and 0.1% SDS, 0.1x SSC, respectively. All washes were performed at 42˚C. For detection of the radioactive signal, the membrane was exposed to an X-ray film (Kodak). For northern blotting of high molecular weight RNA, total RNA was separated by denaturing agarose gel electrophoresis. The RNA was transferred to a nylon membrane using the turboblotter system (Whatman) according to the manufacturer's recommendations and crosslinked to the membrane using UV irradiation. Probe hybridization, washing of the membrane, and detection of the radioactive signal was carried out as described for the small RNA northern blot. Sequences of northern blot probes were:

nSINV-7903+	GGTTGCTTCTTCTTCTTCTCCTGCGTTT
nSINV-7940+	AGTGCCATGCGCTGTCTCTTTCCGGGTTTG
nSINV-7969+	TCGAACAATCTGTCGGCCTCCAACTTAA
nSINV-8040+	GCAGAGGTTTCATTACCTTTCCTTCCAT
nMir2940-3p	AGTGATTTATCTCCCTGTCGAC
nTF345-1570+	CTTGCTGGATTTCCGTCCCTTCGCTGATC
nTF345-1630+	AACGAAACGACGTTTGCTTTGGACACTGT
nTF345-1058-	AGAAGCGCTATTCCACGCTCACCATCGCA
nActin	ATGGGCACGGTGTGGGAGACACCA
nRPL5	GCTTCTGCAGGATGCGGCGGGCAA
nTRNA-lys	AAAAGTCCAACGCTCTACCGACTGAGCTACCCGGGC
nU3	AAACTTGCCTACAGAAATGATCCTGTGAAGCACAGT

qRT-PCR for AGO/PIWI proteins

Total RNA was DNaseI-treated (Ambion) and reverse-transcribed using the Taqman Reverse transcription kit (Roche) and random primers following the manufacturer's instructions. qPCR reactions were prepared using GoTaq qPCR SYBR Mastermix (Promega) and measured on a Light Cycler 480 (Roche). Expression of AGO/PIWI genes was internally normalized against the expression of Lysosomal Aspartic Protease (LAP) and the relative mRNA abundance was determined using to the ΔΔCt method (3). The following primers were used for qPCR.

qFw-Piwi1/3	GGCCGTTAGCGAGTCTCAT
qRe-Piwi1/3	GGCAGAACCTTCGTGGTAAG
qFw-Piwi2	CCGCGGGTACACCGCCGTCAACTT
qRe-Piwi2	CGCTGGTCGAACTCGATGCCCCGC
qFw-Piwi4	TCTTCTTCTCCACCACAGCC
qRe-Piwi4	ATGGTGACCACCTCACAGTTAC
qFw-Piwi5	ACGGCATCACATCGAGACTC
qRe-Piwi5	CGACCTCCACGCTGTCCTC
qFw-Piwi6	TTTTCTTCCACCCCGAGCAG
qRe-Piwi6	AATACATTTGCGATGCGGCC
qFw-Piwi7	ATGCGACGAAACTTCAACTTG
qRe-Piwi7	CCAGCAGCAACCGCATAATT
qFw-Ago3	CTCCAGACGACGGTTTTGGA
qRe-Ago3	GCAGGTACGAAATTGGCTGC
qFw-Ago2	ATTTGGCTCAAGATCAACGC
qRe-Ago2	GAGATCGTATGAAGCGGCCA
qFw-Ago1	CGAACAGCATGATGGAAGTG
qRe-Ago1	AAATTGTTTGCCTCGCATGT
qFw-LAP	GTGCTCATTCACCAACATCG
qRe-LAP	AACTTGGCCGCAACAAATAC

Immunoprecipitation

Aag2 cells expressing V5-3xFlag-tagged PIWI proteins were lysed in lysis buffer (50mM Tris-HCl (pH7.8), 150mM NaCl, 1mM EDTA, 1mM DTT, 0.5% NP40, 1x Protease inhibitors). The lysates were cleared by incubation with washed Protein G agarose beads (Thermo Scientific) at 4˚C under constant rotation for 6 hours. For V5-IP, the cleared lysates were incubated with washed V5 agarose beads (Sigma) overnight under the same conditions. The beads were then washed 5 times in wash buffer I (50mM Tris-HCl (pH7.8), 150mM NaCl, 1mM EDTA), followed by two wash steps in wash buffer II (25mM Tris-HCl (pH7.8) 150mM). Finally, the bound RNA was isolated from the beads using Isol-RNA Lysis Reagent.

Immunofluorescence and western blot analyses

For subcellular localization of PIWI proteins, uninfected or SINV-infected Aag2 cells expressing 3xHA-tagged Piwi5 or Ago3 were fixed on coverslips using 4% paraformaldehyde. Cells were permeabilized in PBS/0.1% Triton and incubated with rabbit anti-HA (1:200 dilution; Abcam ab 9110), or mouse anti-dsRNA (1:1000 dilution; English & Scientific consulting J2 mAb) antibodies. Subsequently, cells were washed in PBS/0.1% Triton and incubated with secondary antibodies (1:400 dilution, goat anti mouse-AlexaFluor594, or goat anti rabbit-AlexaFluor488; Life technologies). After

washing, the nuclei were stained with Hoechst reagent and cover slips were fixed to microscope slides using Mowiol. Pictures were taken on an Olympus FV1000 confocal microscope.

For western blot, proteins were size separated on SDS-PAGE gels and transferred to a nitrocellulose membrane. Antibodies used to detect the proteins were: mouse anti-Flag M2 (1:1000 dilution, Sigma), rat anti-tubulin alpha (1:1000 dilution, Sanbio), and mouse anti-H3K9me2 (1:1000 dilution, Abcam ab1220). Secondary antibodies were IRdye680 or IRdye800 conjugated goat anti mouse or goat anti rat, respectively (both 1:15000 dilution; Li-cor).

5’ RACE and Piwi5 RT-PCR

5’RACE for Piwi5 was performed using the First Choice RNA Ligase Mediated RACE kit (Ambion) according to the manufacturer’s instructions. Briefly, total RNA was treated with Calf Intestine Alkaline Phosphatase (CIP) to remove free 5’ phosphate groups and Tobacco Acid Pyrophosphatase (TAP) to remove the cap of mRNAs. The RNA was then ligated to the 5’RACE RNA adapter, reverse transcribed and PCR amplified. The PCR product was sequenced by Sanger technology. Piwi5 RT-PCR was performed on Aag2 cDNA using Thermoperfect DNA Polymerase. The following primers were used:

pFw-Piwi5A	ACGGCATCACATCGAGACTC (=qFw-Piwi5)
pFw-Piwi5B	CAGCAACCGCAACAGCCAGCGCCT
pRe-Piwi5C	GTGCTTCTCCGCCAGTGGCACCCC
pRe-Piwi5D	AAATGCTCCAAGCGCTGTAT

SUPPLEMENTARY REFERENCES

1. Vodovar, N., Bronkhorst, A.W., van Cleef, K.W., Miesen, P., Blanc, H., van Rij, R.P. and Saleh, M.C. (2012) Arbovirus-derived piRNAs exhibit a ping-pong signature in mosquito cells. *PloS one*, **7**, e30861.
2. Pall, G.S. and Hamilton, A.J. (2008) Improved northern blot method for enhanced detection of small RNA. *Nature protocols*, **3**, 1077-1084.
3. Livak, K.J. and Schmittgen, T.D. (2001) Analysis of relative gene expression data using real-time quantitative PCR and the 2(-Delta Delta C(T)) Method. *Methods*, **25**, 402-408.
